# Systems Based Study of the Therapeutic Potential of Small Charged Molecules for the Inhibition of IL-1 Mediated Cartilage Degradation

**DOI:** 10.1371/journal.pone.0168047

**Published:** 2016-12-15

**Authors:** Saptarshi Kar, David W. Smith, Bruce S. Gardiner, Alan J. Grodzinsky

**Affiliations:** 1 School of Computer Science and Software Engineering, University of Western Australia, Crawley, WA, Australia; 2 Department of Physics and Nanotechnology, Murdoch University, Murdoch, WA, Australia; 3 Department of Biological Engineering, Massachusetts Institute of Technology, Cambridge, MA, United States of America; Universite de Nantes, FRANCE

## Abstract

Inflammatory cytokines are key drivers of cartilage degradation in post-traumatic osteoarthritis. Cartilage degradation mediated by these inflammatory cytokines has been extensively investigated using *in vitro* experimental systems. Based on one such study, we have developed a computational model to quantitatively assess the impact of charged small molecules intended to inhibit IL-1 mediated cartilage degradation. We primarily focus on the simplest possible computational model of small molecular interaction with the IL-1 system—direct binding of the small molecule to the active site on the IL-1 molecule itself. We first use the model to explore the uptake and release kinetics of the small molecule inhibitor by cartilage tissue. Our results show that negatively charged small molecules are excluded from the negatively charged cartilage tissue and have uptake kinetics in the order of hours. In contrast, the positively charged small molecules are drawn into the cartilage with uptake and release timescales ranging from hours to days. Using our calibrated computational model, we subsequently explore the effect of small molecule charge and binding constant on the rate of cartilage degradation. The results from this analysis indicate that the small molecules are most effective in inhibiting cartilage degradation if they are either positively charged and/or bind strongly to IL-1α, or both. Furthermore, our results showed that the cartilage structural homeostasis can be restored by the small molecule if administered within six days following initial tissue exposure to IL-1α. We finally extended the scope of the computational model by simulating the competitive inhibition of cartilage degradation by the small molecule. Results from this model show that small molecules are more efficient in inhibiting cartilage degradation by binding directly to IL-1α rather than binding to IL-1α receptors. The results from this study can be used as a template for the design and development of more pharmacologically effective osteoarthritis drugs, and to investigate possible therapeutic options.

## Introduction

In this paper, we model both IL-1 driven degradation of cartilage explants and the ability of selected small molecule inhibitors (MW 3 to 10 kDa) to modify this tissue response. Our goal is to build a quantitative understanding of IL-1 mediated cartilage degradation in the presence of electrically charged small molecules intended to reduce IL-1 induced cartilage degradation. To this end, we have developed an extended version of our previously experimentally validated computational model used for simulating IL-1α mediated degradation of cartilage tissue [1]. Our previous model simulated the transport of IL-1α, the interaction between IL-1α and its receptors (IL-1R) on the surface of the chondrocytes, secretion of aggrecanases (ADAM-TS4 and ADAM-TS5) and matrix metalloproteinases (MMP-1 and MMP-13) by chondrocytes and the degradation of aggrecan and collagen [1]. The computational model developed in this study includes all these biochemical interactions, and the interaction of the small molecule with IL-1 or its receptor. However, this model also takes into account: (i) the negative fixed charged on cartilage tissue, (ii) the physiological ionic strength of the support medium (*in vitro*)/synovial fluid (*in vivo*), (iii) the electrical charge on IL-1α and selected drugs (small molecule inhibitors) and (iv) Donnan partitioning of electrically charged molecules between support medium/synovial fluid and cartilage tissue. Our primary purpose is to develop a computation model for analysing experimental data and investigating hypotheses related to IL-1 driven degradation of cartilage extracellular matrix.

The interleukins comprise a large group of molecules that play key roles in the regulation of inflammation and innate immunity [2]. Within the interleukins, the IL-1 family of biomolecules plays a central role [3]. The IL-1 family has many components, including seven pro-inflammatory agonists (IL-1α, IL-1β, IL-18, IL-33, IL-36α, IL-36β and IL-36γ), three receptor antagonists (IL-1Ra, IL-36Ra, IL-38) and an anti-inflammatory cytokine (IL-37) [3, 4]. The IL-1 family interacts with the IL-1 receptor (IL-1R) family of biomolecules [4], which itself includes four signaling receptor complexes, two decoy receptors (IL-1R2, IL-18BP) and two negative regulators (TIR8 or SIGIRR, IL-1RAcPb) [2, 4]. As is usual for most biological signaling systems, the complexity of this system helps to ensure that the right balance is struck over time between cellular unresponsiveness to an IL-1 signal and excessive amplification of any signal [4].

When a signaling imbalance does lead to excessive inflammation [4], molecular modifiers are sought that either restrict or enhance interactions within the IL-1 signaling system, which offer hope of a therapeutic benefit [5, 6]. For example, the most important current inhibitors of IL-1 signaling include canakinumab (a monoclonal antibody that binds to IL-1β), anakinra (an IL-1R antagonist), rilanocept (a decoy IL-1α and IL-1β receptor) and gevokizumab (a monoclonal antibody that allosterically regulates IL-1β) [5, 7, 8]. However, canakinumab, rilanocept and gevokizumab are all large molecules (MW = 145–200 kDa) [9–11], which leads to steric exclusion from some tissues including cartilage—the tissue of interest in this study [12]. In contrast, anakinra has a molecular size similar to IL-1 (MW = 17 kDa) [13], and so it can access cartilage tissue.

Given the role of inflammatory mediators in driving tissue damage in post-traumatic joint injury [1, 14, 15], and the possibility that anakinra may significantly modify such inflammatory states, a single dose pilot intervention clinical trial of anakinra was undertaken on people within 30 days of post-traumatic knee joint injury [15]. However the clinical trial found no significant difference between treatment and control groups across 21 biomarkers measured in both serum and synovial fluid [15]. The authors concluded that the absence of a statistically significant outcome was partly attributable to the smaller sampling size of the study (eleven patients). However, the more important factor was believed to be the short *in vivo* half-life of anakinra (four to six hours) following the single intra-articular injection [15, 16]. Most importantly, the outcome of this clinical trial designed to modify IL-1 signaling highlights the importance of first understanding the pharmacokinetics and pharmacodynamics of potential drugs targeted to particular tissues. Indeed, the computational model described herein is specifically designed to quantitatively model IL-1 mediated degradation of cartilage in the presence of a drug that interacts with IL-1 or IL-1R.

Unfortunately, there is very little information in literature about the systematic cataloguing of these desirable small molecular properties to target specific tissues. In the 1990’s tests were conducted on very small molecules (MW < 500) to find suitable candidates to block the actions of IL-1, 2, and 5, but without much success [17]. The failure of these tests was attributed to the size of the small molecules tested (MW < 500 Da) compared to the target cytokines and their corresponding receptors (MW = 8 kDa to 80 kDa) [18–20]. Typically, cytokines and their receptors have a larger ‘interaction surface’, with binding dissociation constants ranging from 1–100 pM [20]. For example, IL-1 (MW = 17 kDa) interacts with high affinity IL-1 receptors (80 kDa transmembrane glycoprotein) with a dissociation constant ranging from 3–8 pM [21]. In contrast, ‘high affinity’ small molecules have their affinity restricted by their small interaction surface, and so their binding dissociation constant typically ranges from 10 to 100 nM [20]. So having comparatively small dissociation constants, at low concentrations most small molecule antagonists are simply outcompeted by native ligands.

The limitations associated with binding of very small molecules to cytokines or its receptors has led to a search of somewhat larger molecules (MW>2 kDa), which have correspondingly larger surface areas and so are more likely to have smaller dissociation constants, and so likely to be therapeutically effective at lower concentrations. Furthermore, small molecules of MW greater than 2 kDa of appropriate shape and binding specificity also offer the potential for the molecule to bind at several spatially discrete sites on the target molecule, particularly if these molecules involve two or more small molecules joined by a ‘linker’ molecule [22]. It has been found that acidic polysaccharides bind well to IL-1, the prototypical molecule in this class being heparin [23, 24]. This has led to a search for glycans that can bind to interleukins. Initial studies in this area have shown promising results [24]. However, an important issue for highly sulphated glycans in cartilage tissue is their negative charge. This highlights the importance of taking into account the Donnan partitioning of any small drug from the synovial fluid into cartilage in the computational model of cartilage degradation.

For simplicity, in this paper we primarily focus on the simplest possible model of small molecular interaction with the IL-1 system, that is, direct binding of the small molecule (SM) to the active site on the IL-1 molecule itself. However, the model is extensible and can be adjusted to include any modifier of the IL-1 cascade, including competitive binding of the SM to the active site on the IL-1 receptor, or binding to downstream products of IL-1 system activation, such as aggrecanases. To illustrate this, here we also change the model so that the SM binds IL-1R competitively (along with IL-1), rather than binding to IL-1 directly. In the following, we first use the model to explore the uptake and release kinetics of a small molecule (MW 3 to 10 kDa) by cartilage tissue. We have selected the molecular weight of the small molecule based on the reported molecular weights of known osteoarthritis inhibitors including sodium/calcium pentosan polysulfate (PPS) [25–27], IGF-1 [28, 29], glucosamine sulfate [30, 31] and chondroitin sulfate [32, 33]. However should the need arise, transport of larger molecules could be modelled by taking into account steric exclusion. We then model the direct interaction of the small molecule with IL-1α, and use the model to predict changes in the rate of cartilage degradation depending on both charge and binding affinity. Then we use the model to explore the effects of changes in the timing of therapeutic intervention following elevation of IL-1α as observed during the early stages of post-traumatic osteoarthritis [15]. Finally, we consider the inhibition of IL-1α mediated cartilage degradation by competition between IL-1α and SM for the binding site on IL-1R. We find that for the same binding constant, the SM is much less effective at inhibiting cartilage degradation when the SM competitively inhibits IL-1R than when it binds directly to IL-1α.

## Materials and Methods

### Model Description

We develop a computational model to simulate the biochemical interaction of a small charged drug molecule (molecular weight assumed to be about 3 to 10 kDa) with articular cartilage tissue, taking into account the charge on the molecule, with valencies ranging from -16 to +16. We have selected the molecular weight and charge range of the small molecule based on values reported in literature for known osteoarthritis (OA) inhibitors including sodium/calcium pentosan polysulfate (PPS) [25–27], IGF-1 [12, 28, 34], glucosamine sulfate [30, 31] and chondroitin sulfate [32, 33, 35]. The model accounts for cation inclusion and anion exclusion in equilibrium for a given negative fixed charge in cartilage. We first use the model to simulate the IL-1α mediated cartilage degradation process. Subsequently, we use the model to demonstrate the kinetics of non-equilibrium transport of the small molecule into cartilage, and we calculate the equilibration time for sorption and desorption of the small molecule in cartilage. We then link this drug model to an IL-1 mediated cartilage degradation model and simulate IL-1 inhibition by the small molecule.

The cartilage degradation model is based on our earlier model of IL-1α mediated biochemical degradation of bovine calf cartilage tissue explants [1], modified to account for the transport of charged species, including sodium ions (Na^+^), chloride ions (Cl^-^), IL-1α and a small charged molecule (SM). The complete model accounts for the kinetics of the chemical interaction between IL-1α and the SM. We first calibrate the model by setting the net charge on IL-1α to -1, and first assume no chemical interaction between IL-1α and the SM. The magnitude of the net charge on IL-1 is selected based on the reported IL-1α isoelectric point (pI) of 6 [36] and assuming a neutral pH of 7.0 (i.e. approximately physiological pH). We then model the equilibrium distribution of the small molecules within cartilage tissue for various net charges on the small molecule. We select net charge magnitudes ranging from -16 to +16. The average basal fixed charge within the cartilage is assumed to be -0.2 mmoles/g of cartilage [37], but the fixed charge is allowed to vary with depth of the cartilage tissue based on the reported *in vivo* depth based variation in aggrecan concentration [1, 38, 39]. For these simulations, we first assume that there is no chemical interaction between the SM and IL-1α. This simulation demonstrates how the electrical charge of the small molecule mediates its sorption and desorption kinetics, independent of any binding interactions. We then extend this model to simulate the uptake and release of positively charged small molecule (SM) by cartilage tissue, taking into account the binding of the molecule to the cartilage ECM, as reported previously [12, 40].

Next we model the inhibitory action of the SM on the biochemical degradation of the cartilage tissue, based on enabling chemical interactions between IL-1α and the SM. The degradation of cartilage is monitored by the release of aggrecan from the cartilage to the surrounding media. This analysis is performed in two distinct steps. In the first step, we calibrated the model by simulating *in vitro* experiments of biochemical degradation of cartilage tissue explants in the presence of the SM inhibitor. For the purposes of illustration, in this step we use experimental data for pentosan polysulfate (PPS) inhibiting cartilage degradation [26], and here assume that the mechanism of action of PPS is by its direct binding to IL-1 (note: by extending this model, we can later change this model assumption as desired). This model calibration procedure demonstrates how to find the kinetic parameters related to the interaction of SM with IL-1α.

Second, we employ this calibrated model to examine the effect of (i) net electrical charge of the SM and (ii) timing of SM administration, on the inhibition of aggrecan and collagen degradation. Additional parametric studies then investigate the effect of changing the dissociation constant related to the interaction between the SM and IL-1α. This serves to generalise the findings of limited experimental data, which is a key advantage of computational modelling.

### Model Geometry

**Fig 1:**
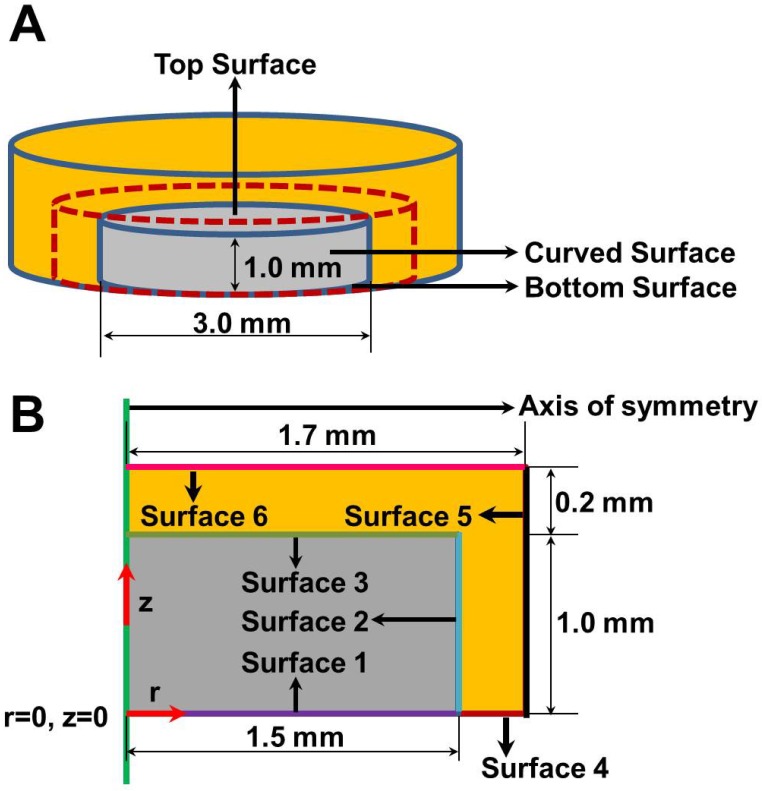
Schematic of cartilage tissue explant and its adjoining environment containing the inflammatory cytokine IL-1α and the small molecule (SM). The inflammatory cytokine IL-1α activates biochemical pathways in the cartilage tissue that promote cartilage degradation. The SM acts to inhibit the biochemical pathways promoting cartilage degradation. **Panel A** shows the actual cylindrical geometry of the explant (grey) cultured in a medium (orange) in the presence or absence of the inflammatory cytokine IL-1α and SM. A cylinder concentric (dotted brown outline) to the cylinder representing the cartilage explant indicates the domain boundary for the computational model. The domain includes both the cartilage tissue explant and a section representing the support medium. The labels indicate the locations of the bottom (surface 1), curved (surface 2) and top (surface 3) of the cartilage tissue explant. **Panel B** shows the two-dimensional (2D) representation of the computational domain. The axis of symmetry is represented by the solid green line. The purple line represents the cartilage tissue explant surface in direct contact with the bottom surface of the well plate (surface 1). The aqua blue and light green lines represents the curved (surface 2) and top (surface 3) of the cartilage tissue explant, respectively. The brown, black and pink lines represent the bottom (surface 4), curved (surface 5) and top (surface 6) surfaces of the domain boundary. The section of the computational domain shaded in grey represents the cartilage tissue explant. The section of the computational domain shaded in orange represents the support medium. The radial and axial co-ordinates of the model geometry are represented by the symbols r and z, respectively. The horizontal and vertical red arrows indicate the positive direction of the r and z co-ordinate axis.

Based on our earlier model of cartilage explant degradation [1], we consider a cylindrical cartilage explant of diameter 3.0 mm and thickness 1.0 mm. This is consistent with the experimental data used for model calibration [14]. We assume radial symmetry along the vertical axis of the explant [1]. Therefore, the cartilage is represented by an axisymmetric two-dimensional spatial domain with radius (r) of 1.5 mm and thickness (z) of 1.0 mm as shown in Fig 1. The current model accounts for the effect of electrical charge on Na^+^, Cl^-^, IL-1α and SM. These species undergo Donnan partitioning between the synovial fluid or support media and cartilage tissue [12, 41, 42], which has a pre-determined fixed charge density. We include the synovial fluid or support media in the model. Assuming radial symmetry, the support medium/synovial fluid is represented by a two-dimensional spatial domain of 0.2 mm thickness (r = 1.5 mm to 1.7 mm and z = 1.0 mm to 1.2 mm) surrounding the cartilage explant as shown in Fig 1. It is to be noted that the governing equations for the non-charged species are only solved over the domain representing the cartilage tissue. In contrast, the equations representing the electrically charged species are solved over both the cartilage tissue and the surrounding support medium/synovial fluid.

### Governing equations

As with our earlier model [1], the current model accounts for the transport of IL-1α and its interaction with the IL-1α receptors present on the surface of the chondrocytes. Proteases secreted through the IL-1α activation of chondrocytes include aggrecanases and matrix metalloproteinases (MMP), which then interact with and degrade the key structural ECM components, aggrecan and collagen. The transport and chemical interactions of the non-charged and the electrically charged species is mathematically represented by the transient Nernst-Planck equation with a source/sink term representing sources or sinks and chemical reactions:

$$\frac{\partial {\text{C}}_{\text{i}}}{\partial \text{t}}={\text{D}}_{\text{i},\text{j}}\nabla^{2}{\text{C}}_{\text{i}}+{\text{Z}}_{\text{i}}{\text{F}\mu }_{\text{i},\text{j}}{\text{C}}_{\text{i}}\nabla^{2}\text{V}\pm {\text{R}}_{\text{i},\text{j}}$$ (1)

In Eq (1), suffix ‘i’ denotes the variables representing the non-charged and electrically charged species in this model. The non-charged species include chondrocyte (‘cell’), intact aggrecan (‘ag’), degraded aggrecan (‘agd’), intact collagen (‘col’), degraded collagen (‘cold’), aggrecanase (‘aga’) and MMP (‘mmp’). The electrically charged species include Na^+^ (‘Na’), Cl^-^ (‘Cl’), IL-1α (‘IL-1’) and SM (‘d’). D_i_ (m^2^/s) and C_i_ (cells/m^3^ or moles/m^3^) represent the effective diffusivity and concentration of the i^th^ species, respectively. The source or sink term, R_i_ (moles/m^3^/s) represents the rates of generation or consumption/apoptosis of the respective species.

The suffix ‘j’ denotes the domains/medium over which the governing equations are solved. The two media applicable to our model include the support medium/synovial fluid (j = f) and the cartilage tissue (j = c). Z_i_ denotes the valency or the magnitude of the net electrical charge of the species ‘i’ and F (C/moles) denotes the Faraday’s constant. For non-charged species, Z_i_ is set to zero, which effectively converts Eq (1) into a transient reactive-diffusive transport partial differential equation. The parameter μ_i,j_ (moles.s/kg) denotes the electrical mobility of species ‘i’ in the j^th^ medium. V (mV) denotes the electrostatic potential. The spatial variation of electrical potential is estimated from the Poisson’s equation, as shown below:

$$\nabla^{2}\text{V}=\frac{-\rho_{\text{j}}}{\varepsilon_{0}\varepsilon_{\text{j}}}$$ (2)

Where ρ_j_ (C.m^-3^) denotes the total space charge density in the j^th^ medium, ε_0_ is the permittivity of free space (ε_0_ = 8.85×10^-12^farad.m^-1^) and ε_j_ is the relative permittivity of the j^th^ medium. We assume the relative permittivity of the two media to be equal to that of water [42]. This assumption is further justified by the fact that approximately 80% of cartilage tissue mass is made up of water [43, 44]. The charge density of the individual media (ρ_f_ and ρ_c_) is calculated according to Donnan theory [12, 42], which assumes partitioning of the mobile charged species between the two phases to achieve electroneutrality in equilibrium. Mathematically, this can be represented as:

$$\rho_{\text{j}}=\text{F}\left(\sum {\text{Z}}_{\text{i}}{\text{C}}_{\text{i}}-\text{FCD}\right)$$ (3)

where FCD represents cartilage fixed charge density, which is primarily a function of the local aggrecan concentration in cartilage [37, 41, 42]. In the support medium/synovial fluid, FCD is assumed to be zero [41, 45]. The governing equations representing the transport and chemical interactions of the individual species inside the cartilage and the support medium/synovial fluid are shown in Table 1 for this modelling study.

**Table 1:** Model governing equations.

Species/Variables	Equation
Chondrocyte (C_cell_)	$\frac{\partial {\text{C}}_{\text{cell}}}{\partial \text{t}}={\text{D}}_{\text{cell}}\nabla^{2}{\text{C}}_{\text{cell}}+{\text{k}}_{1}{\text{C}}_{\text{cell}}-{\text{k}}_{2}{\text{C}}_{\text{cell}}$
Aggrecan (intact) (C_ag_)	$\frac{\partial {\text{C}}_{\text{ag}}}{\partial \text{t}}={\text{D}}_{\text{ag}}\nabla^{2}{\text{C}}_{\text{ag}}+{\text{R}}_{1}{\text{C}}_{\text{cell}}\left(1-\frac{{\text{C}}_{\text{ag}}}{{\text{C}}_{\text{tar}}}\right)-{\text{k}}_{3}{\text{C}}_{\text{aga}}\left(\frac{{\text{C}}_{\text{ag}}}{{\text{C}}_{\text{ag}}+{\text{K}}_{\text{m},\text{aga}}}\right)$
Aggrecan (degraded) (C_agd_)	$\frac{\partial {\text{C}}_{\text{agd}}}{\partial \text{t}}={\text{D}}_{\text{agd}}\nabla^{2}{\text{C}}_{\text{agd}}+{\text{k}}_{3}{\text{C}}_{\text{aga}}\left(\frac{{\text{C}}_{\text{ag}}}{{\text{C}}_{\text{ag}}+{\text{K}}_{\text{m},\text{aga}}}\right)$
Stimulus aggrecanase (S_1_)	$\frac{\partial {\text{S}}_{1}}{\partial \text{t}}=\alpha_{1}\left({\text{C}}^{*}-{\text{S}}_{1}\right)$
Aggrecanase (C_aga_)	$\frac{\partial {\text{C}}_{\text{aga}}}{\partial \text{t}}={\text{D}}_{\text{aga}}\nabla^{2}{\text{C}}_{\text{aga}}+{\text{k}}_{4}{\text{S}}_{1}-{\text{k}}_{5}{\text{C}}_{\text{aga}}$
Collagen (intact) (C_col_)	$\frac{\partial {\text{C}}_{\text{col}}}{\partial \text{t}}={\text{D}}_{\text{col}}\nabla^{2}{\text{C}}_{\text{col}}-{\text{k}}_{\text{act},\text{mmp}}{\text{k}}_{8}{\text{C}}_{\text{mmp}}\left(\frac{{\text{C}}_{\text{col}}}{{\text{C}}_{\text{col}}+{\text{K}}_{\text{m},\text{mmp}}}\right)$
Collagen (degraded) (C_cold_)	$\frac{\partial {\text{C}}_{\text{cold}}}{\partial \text{t}}={\text{D}}_{\text{cold}}\nabla^{2}{\text{C}}_{\text{cold}}+{\text{k}}_{\text{act},\text{mmp}}{\text{k}}_{8}{\text{C}}_{\text{mmp}}\left(\frac{{\text{C}}_{\text{col}}}{{\text{C}}_{\text{col}}+{\text{K}}_{\text{m},\text{mmp}}}\right)$
Stimulus MMP (S_2_)	$\frac{\partial {\text{S}}_{2}}{\partial \text{t}}=\alpha_{2}\left({\text{C}}^{*}-{\text{S}}_{2}\right)$
MMP (C_mmp_)	$\frac{\partial {\text{C}}_{\text{mmp}}}{\partial \text{t}}={\text{D}}_{\text{mmp}}\nabla^{2}{\text{C}}_{\text{mmp}}+{\text{k}}_{10}{\text{S}}_{2}-{\text{k}}_{11}{\text{C}}_{\text{mmp}}{\text{C}}_{\text{cold}}{\text{n}}_{\text{R},\text{cold}}$
Sodium ion (C_Na_)	$\frac{\partial {\text{C}}_{\text{Na}}}{\partial \text{t}}={\text{D}}_{\text{Na}}\nabla^{2}{\text{C}}_{\text{Na}}+{\text{Z}}_{\text{Na}}{\text{F}\mu }_{\text{Na}}{\text{C}}_{\text{Na}}\nabla^{2}\text{V}$
Chloride ion (C_Cl_)	$\frac{\partial {\text{C}}_{\text{Cl}}}{\partial \text{t}}={\text{D}}_{\text{Cl}}\nabla^{2}{\text{C}}_{\text{Cl}}+{\text{Z}}_{\text{Cl}}{\text{F}\mu }_{\text{Cl}}{\text{C}}_{\text{Cl}}\nabla^{2}\text{V}$
IL-1 (C_IL-1_)	$\frac{\partial {\text{C}}_{\text{IL}-1}}{\partial \text{t}}={\text{D}}_{\text{IL}-1}\nabla^{2}{\text{C}}_{1\text{L}-1}+{\text{Z}}_{\text{IL}-1}{\text{F}\mu }_{\text{IL}-1}{\text{C}}_{\text{IL}-1}\nabla^{2}\text{V}-{\text{k}}_{6}{\text{C}}_{\text{IL}-1\text{R}}\left(\frac{{\text{C}}_{\text{IL}-1\alpha }}{{\text{C}}_{\text{IL}-1\alpha }+{\text{K}}_{\text{m},\text{IL}-1}}\right)-{\text{k}}_{7}{\text{C}}_{\text{IL}-1}-{\text{k}}_{12}{\text{C}}_{\text{IL}-1}\left(\frac{{\text{C}}_{\text{d}}}{{\text{C}}_{\text{d}}+{\text{K}}_{\text{m},\text{d}}}\right)$
SM (C_d_)	$\frac{\partial {\text{C}}_{\text{d}}}{\partial \text{t}}={\text{D}}_{\text{d}}\nabla^{2}{\text{C}}_{\text{d}}+{\text{Z}}_{\text{d}}{\text{F}\mu }_{\text{d}}{\text{C}}_{\text{d}}\nabla^{2}\text{V}-{\text{k}}_{12}{\text{C}}_{\text{IL}-1}\left(\frac{{\text{C}}_{\text{d}}}{{\text{C}}_{\text{d}}+{\text{K}}_{\text{m},\text{d}}}\right)$
Electrostatic Potential (V)	$\nabla^{2}\text{V}=-\frac{\rho_{\text{j}}}{\varepsilon_{0}\varepsilon_{\text{j}}}$

### Initial and Boundary Conditions

The initial conditions are shown in Table 2. For intact aggrecan (ag), the initial concentration in the cartilage tissue is set as a function of the tissue thickness (see Table 2). This is based on the reported steady state (turnover rates) *in vivo* aggrecan concentration profiles for young bovine cartilage explants from computational [1, 39] and experimental [38] studies. We set the initial concentrations of Na^+^ and Cl^-^ ions at the reported [41, 42] physiological ionic concentration of 150 moles/m^3^ in both the cartilage tissue and support medium/synovial fluid.

**Table 2:** Model initial conditions.

Species	Values/ Expression (Units)	References
Chondrocyte (C_cell_)	1.5×10^14^ (cells/m^3^)	[46]
Intact aggrecan (C_ag_) (*in vitro*)	$-0.0433{\left(\frac{\text{z}}{\text{H}}\right)}^{4}+0.0431{\left(\frac{\text{z}}{\text{H}}\right)}^{3}-0.0162{\left(\frac{\text{z}}{\text{H}}\right)}^{2}+0.002\left(\frac{\text{z}}{\text{H}}\right)+0.0242\;\left(moles/m^{3}\right)$	[1, 38]
Degraded aggrecan (C_agd_)	0 (moles/m^3^)	[1]
Aggrecanase (C_aga_)	0 (moles/m^3^)	[1]
Intact collagen (C_col_)	0.2 (moles/m^3^)	[1, 43, 47]
Degraded collagen (C_cold_)	0 (moles/m^3^)	[1]
MMP (C_mmp_)	0 (moles/m^3^)	[1]
Sodium ion (C_Na_)	150 (moles/m^3^)	[41, 42]
Chloride ion (C_Cl_)	150 (moles/m^3^)	[41, 42]
IL-1α (C_IL-1_)	0 (moles/m^3^)	[1]
SM (C_d_)	0 (moles/m^3^)	Text
Electrostatic Potential (V)	0 (mV)	Text

The boundary conditions are listed in Table 3. The boundary conditions assigned to the non-charged species are similar to our previous model [1] and include: (i) zero flux boundary condition at the bottom surface (denoted surface 1) of the tissue explant, (ii) zero flux boundary condition at the curved (denoted surface 2) and top (denoted surface 3) surfaces of the tissue explant for chondrocytes (cell) and intact collagen (col), (iii) Robin boundary conditions for intact aggrecan (ag), degraded aggrecan (agd) and MMP (mmp) along the curved and top surfaces of the tissue explant and (iv) Dirichlet type boundary condition for representing the concentrations of aggrecanase (aga) and degraded collagen (cold) at the curved and top surfaces of the explant. A Dirichlet type boundary condition is used to represent the concentrations of the electrically charged species (Na^+^, Cl^-^, IL-1α and SM) at the outer boundaries of the domain (denoted as surface 4, 5 and 6) representing the support medium/synovial fluid. The concentration of the charged species at these locations equals their bulk concentration in the plasma. Similar to our earlier model [1], zero flux boundary condition is applied at the bottom surface of the tissue explant (denoted surface 1) for all the electrically charged species.

**Table 3:** Model boundary conditions.

Species	Surface	Mathematical Expression
Chondrocyte	1, 2, 3	D_cell_∇C_cell_ = 0
Intact aggrecan	1	D_ag_∇C_ag_ = 0
Intact aggrecan	2	D_ag_∇C_ag_ + h_r,ag_ (C_ag_ − C_ag,b_)= 0
Intact aggrecan	3	D_ag_∇C_ag_ + h_z,ag_ (C_ag_ − C_ag,b_)= 0
Degraded aggrecan	1	D_agd_∇C_agd_ = 0
Degraded aggrecan	2	D_agd_∇C_agd_ + h_z,agd_ (C_agd_ − C_agd,b_)= 0
Degraded aggrecan	3	D_agd_∇C_agd_ + h_z,agd_ (C_agd_ − C_agd,b_)= 0
Aggrecanase	1	D_aga_∇C_aga_ = 0
Aggrecanase	2, 3	C_aga_ = 0
Intact collagen	1, 2, 3	D_col_∇C_col_ = 0
Degraded collagen	1	D_cold_∇C_cold_ = 0
Degraded collagen	2, 3	C_cold_ = 0
MMP	1	D_mmp_∇C_mmp_ = 0
MMP	2	D_mmp_∇C_mmp_ + h_r,mmp_ (C_mmp_ − C_mmp,b_) = 0
MMP	3	D_mmp_∇C_mmp_ + h_z,mmp_ (C_mmp_ − C_mmp,b_) = 0
Sodium ion	1	D_Na,c_∇C_Na_ + Z_Na_μ_Na,c_FC_Na_∇V = 0
Sodium ion	4, 5, 6	C_Na_ = C_Na,b_
Chloride ion	1	D_Cl_∇C_Cl_ + Z_Cl_μ_Cl_FC_Cl_∇V = 0
Chloride ion	4, 5, 6	C_Cl_ = C_Cl,b_
IL-1α	1	D_IL-1,c_∇C_IL-1_ + Z_IL−1_μ_IL−1,c_FC_IL−1_∇V = 0
IL-1α	4, 5, 6	C_IL−1_ = C_IL−1,b_
SM	1	D_d_∇C_d,c_ + Z_d_μ_d,c_FC_d_∇V = 0
SM	4, 5, 6	C_d_ = C_d,b_
Electrostatic Potential	1	∇V = 0
Electrostatic Potential	4, 5, 6	V = 0

For the Poisson’s equation, we assume that all the outer boundaries of the computational domain representing the support medium/synovial fluid (denoted surface 4, 5 and 6) are electrically grounded (V = 0). The bottom surface of the tissue explant (denoted surface 1) is considered to be electrically insulated. This is because the resting surfaces of *in vitro* cartilage explants cultured in well plates appear to be in direct contact with the plastic well-plate as evidenced from histological images of cartilage tissue explants incubated in the presence of inflammatory cytokines including IL-1α and TNF-α [1, 48, 49]. In certain simulations, some of the above boundary conditions are rendered time-dependent. The time dependency is introduced to replicate specific experimental/physiological scenarios simulated by the model. Details related to specific modifications introduced in the boundary conditions are discussed in the results section as applicable.

### Model Parameters

Model parameters are listed in Table 4. The majority of the parameters are sourced from our earlier computational model for IL-1α mediated cartilage biochemical degradation [1]. These include i) cartilage explant dimensions, ii) rates of formation and apoptosis of chondrocytes, iii) rates of intact aggrecan synthesis by chondrocytes, iv) Michaelis-Menten kinetic parameters to represent the catalytic activity of proteases (aggrecanase and MMP), v) rates of formation of aggrecanase and MMP including parameters from rate equations capturing the time delay in protease expression, vi) parameters related to the binding kinetics of IL-1α to IL-1 receptors (IL-1R) located on the surface of the chondrocytes and vii) parameters representing the dependence of aggrecan concentration on MMP activity [1, 50].

**Table 4:** Model Parameters.

Parameters	Values/Expressions	References
Effective diffusivity (Chondrocyte) (D_cell_)	0 m^2^/s	[51]
Effective diffusivity (Intact aggrecan) (D_ag_)	1×10^−14^ m^2^/s	[52]
Effective diffusivity (Degraded aggrecan) (D_agd_)(C_ag_ is in moles/m^3^)	${\text{D}}_{\text{agd}}={\text{D}}_{\text{agd}}^{*}{\text{e}}^{-{95\text{C}}_{\text{ag}}}$	[1, 53]
Diffusivity (Degraded aggrecan) (D*_agd_)	1×10^−10^ m^2^/s	[52, 54]
Effective diffusivity (Aggrecanase) (D_aga_)	${\text{D}}_{\text{aga}}={\text{D}}_{\text{aga}}^{*}{\text{e}}^{-{120\text{C}}_{\text{ag}}}$	[1, 53]
Diffusivity (Aggrecanase) (D*_aga_)	1×10^−12^ m^2^/s	[55]
Effective diffusivity (Intact collagen) (D_col_)	0 m^2^/s	[54]
Effective diffusivity (Degraded collagen) (D_cold_)	${\text{D}}_{\text{cold}}={\text{D}}_{\text{cold}}^{*}{\text{e}}^{-{95\text{C}}_{\text{ag}}}$	[1, 53]
Diffusivity (Degraded collagen) (D*_cold_)	1×10^−10^ m^2^/s	[52, 56]
Effective diffusivity (MMP) (D_mmp_)	${\text{D}}_{\text{mmp}}={\text{D}}_{\text{mmp}}^{*}{\text{e}}^{-{95\text{C}}_{\text{ag}}}$	[1, 53]
Diffusivity (MMP) (D*_mmp_)	1×10^−12^ m^2^/s	[55, 57]
Effective diffusivity (Na^+^) (D_Na,c_) (cartilage)	1.33×10^−9^ m^2^/s	Text, [58]
Effective diffusivity (Na^+^) (D_Na,f_) (fluid)(fluid represents support medium or synovial fluid)	1.33×10^−9^ m^2^/s	Text, [58]
Effective Diffusivity (Cl^-^) (D_Cl,c_) (cartilage)	2.03×10^−9^ m^2^/s	Text, [58]
Effective Diffusivity (Cl^-^) (D_Cl,f_) (fluid)	2.03×10^−9^ m^2^/s	Text, [58]
Effective diffusivity (IL-1α) (D_IL-1,c_) (cartilage)	${\text{D}}_{\text{IL-}1}={\text{D}}_{\text{IL-}1}^{*}{\text{e}}^{-{95\text{C}}_{\text{ag}}}$	[1, 53]
Diffusivity (IL-1α) (D*_IL-1_)	7×10^−11^ m^2^/s	[59]
Effective diffusivity (IL-1α) (D_IL-1,f_) (fluid)	7×10^−9^ m^2^/s	text
Effective Diffusivity (SM) (D_d,c_) (cartilage)	2.66×10^−10^ m^2^/s	[12]
Effective Diffusivity (SM) (D_d,f_) (fluid)	2.66×10^−8^ m^2^/s	[12]
Basal aggrecan production rate (R_1_)	${\text{P}}_{\text{ag}}\left(1-\frac{0.9\text{z}}{\text{H}}\right)$	[1]
Target aggrecan (intact) concentration (C_tar_)	0.024 moles/m	[1, 39]
Chondrocyte based basal aggrecan production (P_ag_)	2.4×10^−22^ moles/cell/s	[60, 61]
Thickness of cartilage tissue explant (H)	1×10^−3^ m	Text, [1]
Chondrocyte production rate (k_1_)	0 cells/s	[1]
Chondrocyte apoptosis rate (k_2_)	0 cells/s	[1]
Catalytic rate constant (aggrecanase) (k_3_)	1.45 s^-1^	Text, [62, 63]
Michaelis constant (aggrecanase) (K_m,aga_)	8.5×10^−5^ moles/m^3^	Text, [63, 64]
Rate constant aggrecanase production (k_4_)	0.83k_6_ s^-1^	[1, 65]
Aggrecanase degradation rate (k_5_)	1×10^−4^ s^-1^	[1, 66]
Turnover number (IL-1α binding to IL-1R) (k_6_)	4.32×10^−5^ s^-1^	[1, 67]
Dissociation constant (IL-1α binding to IL-1R) (K_m,IL-1_)	7.2×10^−8^ moles/m^3^	[1, 67]
IL-1α degradation rate (k_7_)	5.83×10^−4^ s^-1^	[1, 68]
IL-1α receptor concentration (C_IL-1R_)	****$\left(\frac{{\text{n}}_{\text{R}}{\text{C}}_{\text{cell}}}{{\text{N}}_{\text{A}}}\right)$****	[69]
Number of IL-1 receptors in single chondrocyte (n_R_)	2700 cell^-1^	[1, 67]
Avogadro number (N_A_)	6.023×10^23^ #/moles	[69]
Na^+^ concentration (bulk fluid/support medium/synovial fluid) (C_Na,b_)	150 moles/m^3^	Text, [41, 42]
Cl^-^ concentration (bulk fluid) (C_Cl,b_)	150 moles/m^3^	Text, [41, 42]
IL-1α concentration (bulk fluid) (C_IL-1,b_)	5.7×10^−8^ moles/m^3^	Text, [14]
SM concentration (bulk fluid) (C_d,b_)	0.0003 moles/m^3^	Text, [26]
Catalytic activity (MMP) (k_8_)	1.5 s^-1^	[70]
Michaelis constant (MMP) (K_m,mmp_)	0.0021 moles/m^3^	[70]
Aggrecan dependent MMP catalytic activity (k_act,mmp_)	$\frac{\beta_{\text{max}}}{1+{\left(\frac{{\text{C}}_{\text{ag}}+{\text{C}}_{\text{agd}}}{{\text{k}}_{9}}\right)}^{\text{n}}}$	[1]
C_ag_+C_agd_ at half-maximal MMP activity (k_9_)	0.0003 moles/m^3^	[1]
Hill–coefficient MMP activity (n)	6	[1]
Maximum MMP activity (β_max_)	1	[1]
Rate constant MMP production (k_10_)	0.17k_6_s^−1^	[1, 65]
Rate constant MMP binding to degraded collagen (k_11_)	0.47 M^-1^.s^-1^	[70]
# of MMP binding sites on degraded collagen (n_R,cold_)	320	[1]
Turnover number (IL-1α binding to SM) (k_12_)	1 s^-1^	Text, [12, 26]
Dissociation constant (IL-1α binding to SM) (K_m,d_)	1×10^−3^ moles/m^3^	Text, [12, 26]
IL-1-IL-1R complex equilibrium concentration (C*)	$\left(\frac{{\text{C}}_{\text{IL-}1}{\text{C}}_{\text{IL-}1\text{R}}}{{\text{C}}_{\text{IL-}1}+{\text{K}}_{\text{m},\text{IL-}1}}\right){\text{moles}/\text{m}}^{3}$	[71]
Rate constant aggrecanase stimulus (α_1_)	0.4×10^−5^ s^-1^	[1]
Rate constant MMP stimulus (α_2_)	0.4×10^−5^ s^-1^	[1]
Intact aggrecan radial mass transfer coefficient (h_r,ag_)	1×10^−10^ m/s	[1]
Intact aggrecan axial mass transfer coefficient (h_z,ag_)	0.8×10^−10^ m/s	[1]
Degraded aggrecan radial mass transfer coefficient (h_r,agd_)	1.5×10^−8^ m/s	[1]
Degraded aggrecan axial mass transfer coefficient (h_z,agd_)	1.2×10^−8^ m/s	[1]
MMP radial mass transfer coefficient (h_r,mmp_)	1×10^−9^ m/s	[1]
MMP axial mass transfer coefficient (h_z,mmp_)	1×10^−9^ m/s	[1]
Synovial fluid intact aggrecan concentration (C_ag,b_)	0 moles/m^3^	[1, 39]
Support medium intact aggrecan concentration (C_ag,b_)	0 moles/m^3^	[1]
Support medium degraded aggrecan concentration (C_agd,b_)	0 moles/m^3^	[1]
Support medium MMP concentration (C_mmp,b_)	0 moles/m^3^	[1]
Cartilage fixed charge density (FCD)	$-\frac{200\left({\text{C}}_{\text{ag}}+{\text{C}}_{\text{agd}}\right)}{0.024}{\text{moles}/\text{m}}^{3}$	Text, [37]
Valency/ charge magnitude (Na^+^) (Z_Na_)	+1	[41]
Valency/ charge magnitude (Cl^-^) (Z_Cl_)	-1	[41]
Valency/charge magnitude (IL-1α) (Z_IL-1_)	-1	[36]
Valency/charge magnitude (SM) (Z_d_)	-16 to +16	Text, [12]
Electrical mobility (general) (μ_i,j_)	$\left(\frac{{\text{D}}_{\text{i},\text{j}}}{\text{RT}}\right)$	[72]
Electrical mobility (Na^+^) (cartilage) (μ_Na,c_)	5.16×10^−13^ s.moles/kg	[41, 72]
Electrical mobility (Na^+^) (fluid) (μ_Na,f_)	5.16×10^−13^ s.moles/kg	[41, 72]
Electrical mobility (Cl^-^) (cartilage) (μ_Cl,c_)	7.8×10^−13^ s.moles/kg	[41, 72]
Electrical mobility (Cl^-^) (fluid) (μ_Cl,f_)	7.8×10^−13^ s.moles/kg	[41, 72]
Electrical mobility (IL-1α) (cartilage) (μ_IL-1,c_)	$\left(\frac{{\text{D}}_{\text{IL}-1,\text{c}}}{\text{RT}}\right)$	[72]
Electrical mobility (IL-1α) (fluid) (μ_IL-1,f_)	2.7×10^−12^ s.moles/kg	[36, 72]
Electrical mobility (SM) (cartilage) (μ_d,c_)	1.03×10^−13^ s.moles/kg	[12, 72]
Electrical mobility (SM) (fluid) (μ_d,f_)	1.03×10^−11^ s.moles/kg	Text, [12, 72]
Permittivity (free space) (ε_0_)	8.85×10^−12^ farad/m	[42]
Relative permittivity (cartilage) (ε_c_)	100×10^8^	Text, [42]
Relative permittivity (fluid) (ε_f_)	100×10^8^	Text, [42]
Faraday’s constant	96500 C/moles	[42]
Universal gas constant (R)	8.314 J/mol/K	[42]
Temperature (T)	310 K	Text
Dissociation constant (SM binding to IL-1R) (K*_m,d_)	1 μM, 1 nM, 100 pM, 1 pM	Text
Dissociation constant (SM binding to ECM) (K_m,ECM_)	100 nM, 1 μM	Text, [12]

Additional model parameters in the current study include those associated with the Nernst-Planck and Poisson’s equations. The parameters include (i) valency or electrical charge magnitude (Z_i_) of the charged species, (ii) electrical mobility of the charged species in cartilage (μ_i,c_) and support medium/synovial fluid (μ_i,f_), (iii) relative permittivity of cartilage tissue (ε_c_) and support medium/synovial fluid (ε_f_), (iv) fixed charge density (FCD) of cartilage tissue and (v) kinetic parameters related to the SM driven inhibition of IL-1α mediated cartilage degradation. As mentioned earlier, we initially assume that the SM inhibits cartilage degradation by directly binding to IL-1α. Hence the rate of binding of IL-1α with the SM is represented by the turnover number, k_12_ (s^-1^) and the dissociation constant, K_m,d_ (moles/^3^). We used K_m,d_ values ranging from 0.001 μM to 1 μM. This was based on reported dissociation constant data related to binding of interleukin family of cytokines with their signaling inhibitors including heparin, canakinumab and rilanocept [5, 24, 73, 74].

Based on the reported FCD’s [37, 75] and density of cartilage tissue (1.15 g/ml) [43], we estimate the basal FCD of cartilage tissue in the range of -150 to -200 moles/m^3^. Shapiro et al. [76] proposed a correlation for calculation of cartilage FCD based on tissue aggrecan concentration. Using the reported steady state maximum *in vivo* aggrecan concentration of 0.024 moles/m^3^ (assuming molecular weight of aggrecan to be 2.5 MDa [77] in young bovine cartilage [1, 38, 39]), we estimate the cartilage FCD to be -233 moles/m^3^. For this study, we select the basal FCD of the cartilage to be -200 moles/m^3^ corresponding to the steady state tissue aggrecan concentration of 0.024 moles/m^3^ and reported mass composition of aggrecan in cartilage tissue [43]. The change in FCD due to spatio-temporal variation in tissue aggrecan concentration is captured using the correlation shown in Table 4.

### Numerical Solution

We solved the governing equations together with the appropriate initial and boundary conditions numerically using the finite element software COMSOL Multiphysics (Version 5.0, Burlington, MA, USA). We performed a mesh dependence study, which showed that beyond 12751 mesh elements, the model predictions are independent of the number of finite elements. We thus used a total of 12751 quadratic finite elements consisting of 917 boundary elements for all our simulations. The relative accuracy for the simulations was set at 1×10^−3^. The simulations were performed on a high end PC (Intel (R) Core i7-4930K CPU @ 3.40 GHz processor with 64 GB RAM) with simulation times ranging from 30–1200 s.

## Results

### Model Calibration

This work builds upon our previous model of IL-1α mediated degradation of bovine calf cartilage explants [1]. However, this study is significantly different from our earlier model as it takes into account Donnan partitioning of charged molecules as boundary conditions at the cartilage-medium interface. This includes incorporating the fixed charge density of cartilage, the physiological ionic strength of the fluid, and the charge on IL-1α and the SM. These modifications are incorporated into the model through the following steps:

1. Selection of pH and charge on IL-1α. Cartilage has a physiological pH slightly above 7 [78]. We estimated the net charge on IL-1α to be -1 using a protein calculator PepCalc (PepCalc, Innovagen AB, Lund, Sweden).
2. Fixed charge is estimated first by theoretical calculation based on two moles of negative charge per mole of chondroitin sulfate disaccharide unit (one sulfate and one carboxyl) [79] and using the reported molecular weight of 502.5 mg/mmole for a chondroitin sulfate disaccharide unit [76, 80]. In terms of the total aggrecan concentration, the fixed charge of the cartilage tissue can be calculated as follows [76]:
  $$\text{FCD}=\frac{-2\left({\text{C}}_{\text{ag}}+{\text{C}}_{\text{agd}}\right)}{502.5}$$ (4)
  where FCD is the fixed charge of the cartilage tissue expressed as mM and C_ag_ and C_agd_ are the concentrations of intact and degraded aggrecan expressed in mg/l. We used Eq (4) to calculate the fixed charge corresponding to the reported range of aggrecan concentration in young bovine cartilage tissue (55–60 mg/ml) [38]. The calculated fixed charge of the cartilage tissue is consistent with reported fixed charge densities for young bovine cartilage tissue [75].
3. Selection of diffusivities. The effective diffusivity of IL-1α in the cartilage tissue is the same as our earlier modeling study [1]. The effective diffusivity of Na^+^, Cl^-^ and SM were obtained from literature [53, 58]. Though the ‘charge partner’ diffusing with the ion or molecule can influence the magnitude of the diffusion coefficient [58], we chose the effective diffusivities of Na^+^, Cl^-^ and SM to be constant. This is not expected to significantly alter the steady state and transient results presented here because Na^+^ and Cl^-^ ion concentrations are much larger than the SM concentration. Additionally, the molecular weight of Na^+^, Cl^-^ and SM is significantly lower compared to aggrecan, collagen, IL-1α and proteases.

**Fig 2:**
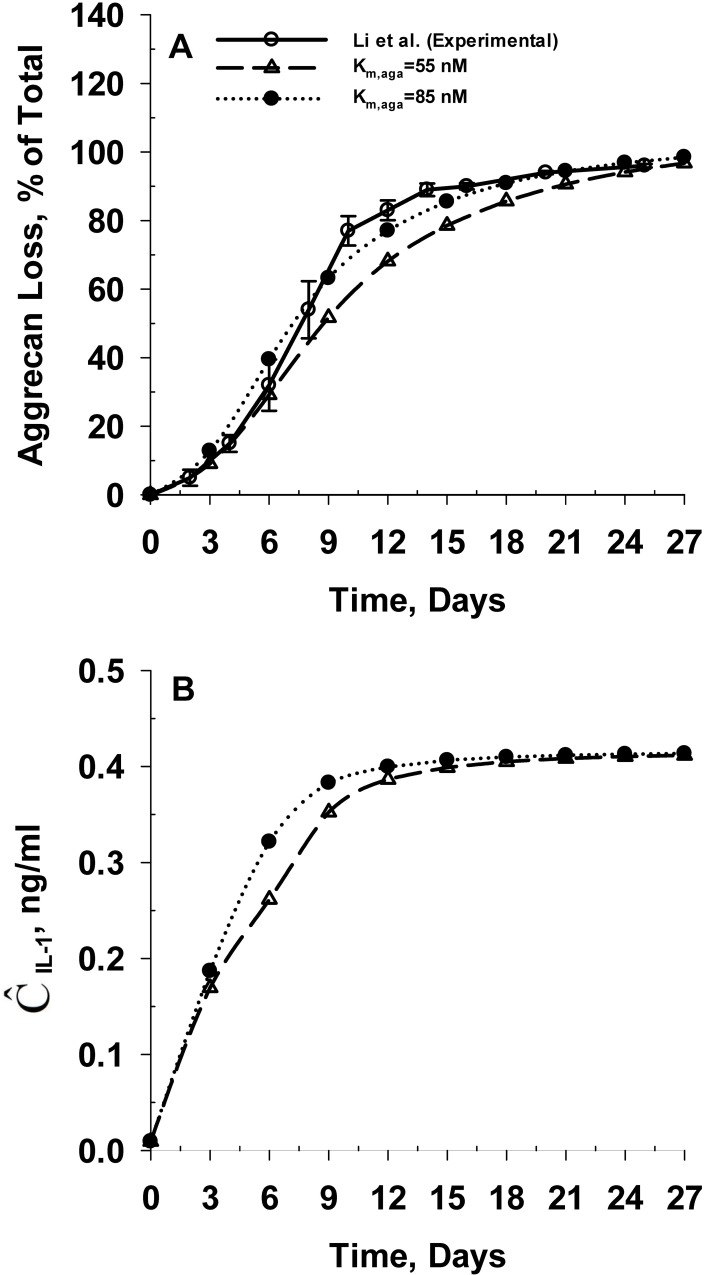
Temporal variation in aggrecan loss from tissue and tissue based IL-1α concentration for young bovine cartilage explants subjected to *in vitro* IL-1α mediated biochemical degradation. **Panel A** shows the comparison between the predicted and experimental [14] rate of aggrecan loss from the explant. **Panel B** shows the temporal variation in the spatial average concentration of IL-1α of the cartilage explant. The model is calibrated by adjusting the Michaelis constant for aggrecanase catalyzed degradation of intact aggrecan (K_m,aga_). The calibrated model accounts for the electrochemical interactions of IL-1α in the support medium and in the cartilage tissue.

We recalibrated the model taking into account Donnan partitioning, by comparing model predictions with the *in vitro* time-dependent aggrecan loss data from young bovine cartilage explants as reported by Li, Wang et al. [14]. These experiments involved culturing of young bovine cartilage explants in adequate support medium both in the absence and presence of the inflammatory cytokine IL-1α for a period of 27 days. Practically, this involved the recalibration of one model parameter (K_m,aga_) to take into account the reduced concentration of IL-1α in the cartilage. The Michaelis constant for aggrecanase catalyzed degradation of intact aggrecan (K_m,aga_) is reported to range from 11.0 nM to 28.2 μM [63, 64, 81]. The predicted aggrecan loss from the explant matched the corresponding experimental data [14] as K_m,aga_ is increased by about 55% from 55 to 85 nM. Hence, the adjusted value of K_m,aga_ remains within its reported range (i.e. 11.0 nM to 28.2 μM [63, 64, 81]). Fig 2A shows the comparison between the experimental [14] and predicted aggrecan loss profile from the explant.

Because IL-1α is negatively charged at physiological pH, ion exclusion means that the concentration for the charged molecule at equilibrium is somewhat lower than for an uncharged molecule at equilibrium. Fig 2B shows the predicted spatial average IL-1α concentration of the explant for the different cases simulated during the model calibration process. Interestingly, the average IL-1α concentration reached a steady-state value of 0.4 ng/ml at 9 days similar to the predictions from our previous model without Donnan partitioning [1]. The steady state average concentration of IL-1α in the explant was considerably less than the concentration in the surrounding media despite ongoing transport into the cartilage. This can be attributed to the rapid protease induced degradation of IL-1α [68] as accounted for in our previous [1] and current model.

### Donnan partitioning of SM

Donnan partitioning leads to the inclusion or exclusion of molecules by the cartilage tissue depending on the fixed charge of the cartilage tissue and net charge of the molecule [12, 80]. We thus used our model to investigate the effect of the SM electrical charge on the: (i) time-dependent uptake of SM from synovial fluid into cartilage tissue and (ii) time-dependent release of SM post-removal of SM from the surrounding media. To study the SM uptake and release kinetics, we simulated a 1D geometry, as the 1D model geometry approximates the *in vivo* drug delivery scenario.

**Fig 3:**
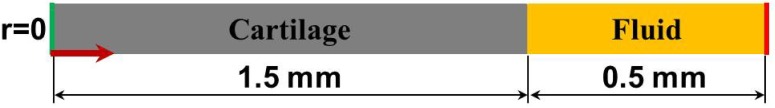
Schematic of the 1D model geometry representing the cartilage tissue and its surrounding media containing synovial fluid. The computational domain includes both the cartilage tissue and a section representing the synovial fluid. The solid red line (outer) represents the outer boundary of the computational domain. The solid green line represents the osteochondral junction (inner). The section of the computational domain shaded in grey represents the cartilage tissue explant. The section of the computational domain shaded in orange represents the synovial fluid. The spatial co-ordinate of the model geometry is represented by the symbol r. The horizontal brown arrow indicates the positive direction of the r co-ordinate axis.

Fig 3 shows the schematic of the model geometry. The depth of the cartilage tissue for the 1D model is set at 1.5 mm, which is consistent with measured *in vivo* depth for young bovine knee cartilage [53, 82]. The concentration of intact aggrecan within the cartilage tissue is set at a spatially-uniform constant value of 60 mg/ml (0.024 moles/m^3^). We solve the governing equations representing the transport of electrically charged species simultaneously with the Poisson’s equation (Eq (3)). For the purposes of simplicity, we assume the molecular weight of the SM to be 3 kDa for all simulations. Additional simulations were performed assuming the molecular weight of the SM as 10 kDa. The effective diffusivity of the small molecule in the cartilage tissue was set at a constant value of 2.6×10^−10^ m^2^/s. This is consistent with measured diffusivities of electrically charged molecules (MW = 3 to 10 kDa) across young bovine cartilage tissue explants [12]. The following boundary conditions are used for the 1D model:

1. We set the concentrations of Na^+^ and Cl^-^ to 150 moles/m^3^ at the outer boundary of the domain representing the synovial fluid (outer). This is based on the reported serum concentration [83] and serum/synovial fluid distribution ratio [84] for sodium chloride. The concentration of the SM is set at 1 μg/ml (i.e. 0.0003 moles/m^3^). This however changes to 0.0001 moles/m^3^ for a 10 kDa molecule.
2. During simulation of the SM release process, the concentration of SM at the outer boundary of the computational domain (outer) is set to zero, while concentrations of Na^+^ and Cl^-^ remain unchanged from the model simulating the SM uptake process.
3. For both the uptake and release simulations, a zero flux boundary condition is set for all the species at the boundary representing the osteochondral junction (inner). The outer boundary of the computational domain representing the synovial fluid (outer) is assumed to be electrically grounded (V = 0 mV), while the boundary representing the osteochondral junction (inner) is assumed to be electrically insulated.

**Fig 4:**
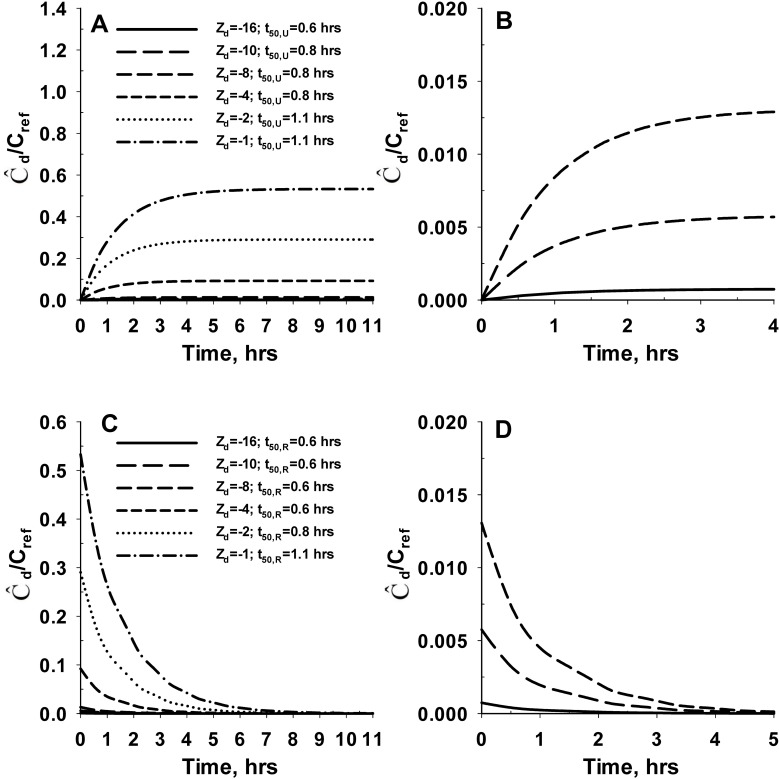
Effect of electrical charge on the *in vivo* uptake and retention of negatively charged SM in cartilage tissue. **Panel A** shows the uptake kinetics of negatively charged SM by the cartilage tissue. **Panel B** represents the enlarged version of Panel A for net SM charge of -16, -10 and -8. The bulk concentration of SM in the plasma (C_d,b_) is assumed to be 1 μg/ml during the uptake process (C_ref_ = 1 μg/ml). **Panel C** shows the release kinetics of negatively charged SM from cartilage tissue into the synovial fluid. **Panel D** represents the enlarged version of Panel C for net SM charge of -16, -10 and -8. The bulk concentration of SM in the plasma (C_d,b_) is assumed to be 0 μg/ml during the release process. Z_d_ denotes the valency or net charge of the SM. The time required for the average SM concentration in the cartilage tissue to reach 50% of the steady-state value during the uptake process is denoted as t_50,U_. The time required for the SM concentration to reach 50% of the initial value during the release process is denoted as t_50,R_. The molecular weight of the SM is assumed to be 3 kDa for these simulations. The concentration of intact aggrecan throughout the cartilage tissue is set at a constant value of 60 mg/ml.

Fig 4 shows the predicted average concentration of SM (Ĉ_d_) in the cartilage tissue for *negatively* charged SM during the uptake (Fig 4A and 4B) and release (Fig 4C and 4D) process. The results show that exposure of the cartilage tissue to the SM leads to an initial period of SM uptake by the cartilage tissue. This is indicated by the time-dependent increase in the average concentration of SM in the cartilage tissue. Subsequently, the average tissue SM concentration reaches a steady-state value indicating equilibration of the electrochemical potential between the cartilage tissue and the synovial fluid. The time required for the average SM concentration in the cartilage tissue to reach 50% of the steady-state value (t_50,U_) range from 0.6 hours (Z_d_ = -16) to 1.1 hrs (Z_d_ = -1). The corresponding steady-state average SM concentration in the cartilage tissue range from 0.0007 μg/ml (Z_d_ = -16) to 0.5 μg/ml (Z_d_ = -1). The results from the desorption simulations show an instantaneous release of SM from the cartilage tissue into the synovial fluid. This is indicated by the time-dependent reduction in the average SM concentration of the cartilage tissue. The release of SM from the cartilage tissue continues till all the SM partitioned from synovial fluid to cartilage tissue during the uptake process is depleted. The time required for the SM concentration to reach 50% of the initial value during the release process (t_50,R_) range from 0.6 hours (Z_d_ = -16) to 1.1 hours (Z_d_ = -1) (i.e. similar to the rate for uptake). The results from this analysis demonstrate that the negative fixed charge of the cartilage results in exclusion of negatively charged molecules (anion) from the cartilage ECM.

**Fig 5:**
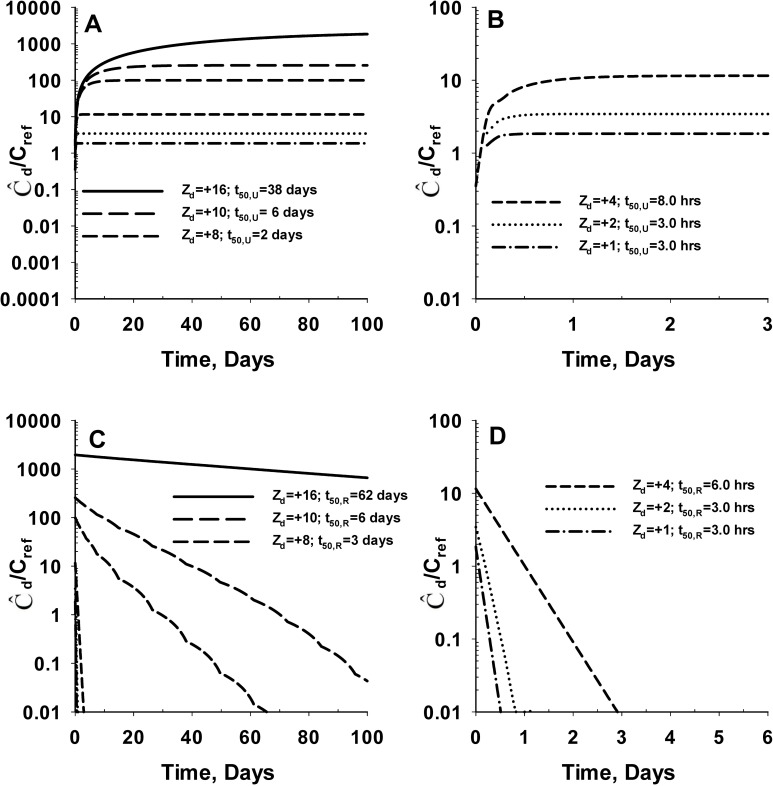
Effect of electrical charge on the *in vivo* uptake and retention of positively charged SM in cartilage tissue. **Panel A** shows the uptake kinetics of positively charged SM by the cartilage tissue. **Panel B** represents the enlarged version of Panel A for net SM charge of +4, +2 and +1. The bulk concentration of SM in the plasma (C_d,b_) is assumed to be 1 μg/ml during the uptake process (C_ref_ = 1 μg/ml). **Panel C** shows the release kinetics of positively charged SM from cartilage tissue into the synovial fluid. **Panel D** represents the enlarged version of Panel C for net SM charge of +4, +2 and +1. The bulk concentration of SM in the plasma (C_d,b_) is assumed to be 0 μg/ml during the release process. Z_d_ denotes the valency or net charge of the SM. The time required for the average SM concentration in the cartilage tissue to reach 50% of the steady-state value during the uptake process is denoted as t_50,U_. The time required for the SM concentration to reach 50% of the initial value during the release process is denoted as t_50,R_. The molecular weight of the SM is assumed to be 3 kDa for these simulations. The concentration of intact aggrecan throughout the cartilage tissue is set at a constant value of 60 mg/ml.

Fig 5 shows the predicted average concentration of SM (Ĉ_d_) in the cartilage tissue for *positively* charged SM during the uptake (Fig 5A) and release (Fig 5B) process. The uptake and release kinetic profiles are similar to the negatively charged SM. However, the predicted equilibration times are considerably longer while the steady-state average SM concentration in cartilage tissue are significantly higher for positively charged SM. The time required for average SM concentration to reach 50% of the steady-state value (t_50,U_) during SM sorption ranged from 3 hours (Z_d_ = +1) to over 38 days (Z_d_ = +16). The corresponding steady-state average SM concentration in the cartilage tissue range from 2.0 μg/ml (Z_d_ = +1) to 1953.4 μg/ml (Z_d_ = +16). The time required for the SM concentration to reach 50% of the initial value during the release process (t_50,R_) range from 3 hours (Z_d_ = +1) to 62 days (Z_d_ = +16). The results from this analysis demonstrate that the negative fixed charge of the cartilage causes inclusion of positively charged molecules into the cartilage ECM (cation inclusion) from synovial fluid. We also note that the time for cation uptake to reach equilibrium is considerably longer than the time required for anion uptake to reach equilibrium.

### Model calibration for inhibition of IL-1α mediated cartilage degradation by the SM

Our next step is to model the inhibition of the IL-1α mediated degradation of the cartilage tissue. To do this, we first assume that the SM inhibits IL-1α mediated cartilage degradation by directly binding to IL-1α. We take into account the mass action reaction kinetics of the SM and IL-1α interaction. K_m,d_ represents the dissociation constant related to binding of IL-1α to SM. We tried three values of the dissociation constant (K_m.d_), namely, 10 nM (Case 1), 100 nM (Case 2) and 1 μM (Case 3). We selected these cases based on reported dissociation constants related binding of interleukin family of cytokines with their corresponding inhibitors [5, 24, 73, 74]. We assume a net charge of -1 on IL-1α (at physiological pH) and -16 on the SM. The molecular weight of the SM is assumed to be 3 kDa.

**Fig 6:**
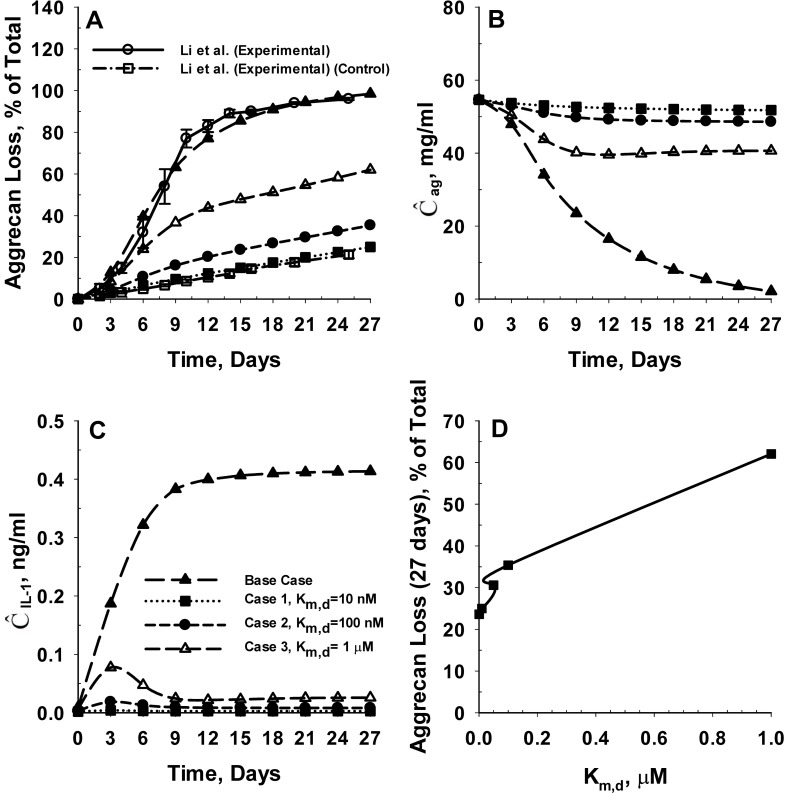
Effect of inhibition of IL-1α mediated degradation of cartilage tissue by SM. **Panel A** shows the effect of the rate of inhibition of IL-1α by SM on the predicted rates of aggrecan losses from the cartilage tissue. The predicted rates of aggrecan loss are compared with *in vitro* aggrecan loss data from young bovine cartilage explants in the absence (control) and presence of IL-1α [14]. K_m,d_ represents the dissociation constant of binding of IL-1α and the SM. The model was simulated at three distinct values of the dissociation constant (K_m.d_) including 10 nM (Case 1), 100 nM (Case 2) and 1 μM (Case 3). The chemical interaction between IL-1α and SM is not accounted for in the ‘Base Case’ scenario. **Panel B** and **Panel C** show the temporal variation in the spatial average intact aggrecan and IL-1α concentration of the cartilage explant for the different cases simulated. **Panel D** shows the effect of K_m,d_ on the extent of aggrecan loss from the cartilage tissue at 27 days after initial exposure to IL-1α. The net charge on IL-1α and SM are assumed to be -1 and -16, respectively. The molecular weight of the SM is assumed to be 3 kDa.

Fig 6A shows the predicted rate of aggrecan loss from cartilage tissue explant for cases 1, 2 and 3, which are compared with the predicted aggrecan loss profile in the absence of chemical interaction between IL-1α and SM (Base Case) (4 curves). For reference, the experimental aggrecan loss data from young bovine cartilage explants in the presence and absence of IL-1α are also shown (2 curves) [14]. Fig 6B and 6C show the spatial average concentration of intact aggrecan (Ĉ_ag_) and IL-1α (Ĉ_IL-1_) for the four cases simulated. The results demonstrate that chemical interaction between SM and IL-1α significantly reduced the IL-1α mediated catabolism of intact aggrecan. In comparison to the base case, the aggrecan loss from the explant is reduced by 75%, 64% and 37% for cases 1 (K_m.d_ = 10 nM), 2 (K_m.d_ = 100 nM) and 3 (K_m.d_ = 1 μM), respectively at 27 days (Fig 6A and 6D).

The predicted aggrecan loss profile from the explant corresponding to K_m,d_ of 1 μM (Case 3) is similar to the reported [26] *in vitro* aggrecan loss from bovine cartilage explants cultured in the presence of 1 μM retinoic acid and 1 μg/ml pentosan polysulfate (PPS) (note: though only the aggrecan loss profiles upon exposure to retinoic acid are shown, the paper says IL-1α has aggrecan loss profiles similar to that of retinoic acid, as confirmed in a later paper by Munteanu et al. [85]). Our results also show that the rate of aggrecan loss from cartilage tissue for case 1 (K_m,d_ = 10 nM) is consistent with reported *in vitro* aggrecan loss data from bovine cartilage explants cultured in the absence of any aggrecan catabolic agents [14, 26] *i*.*e*. a K_m,d_ = 10 nM results in a similar profile to the control case, even when exposed to IL-1α [14]. The computational modelling shows that the steady-state average IL-1α concentration of the cartilage tissue reduced by 99%, 98% and 94% corresponding to case 1 (K_m,d_ = 10 nM), case 2 (K_m,d_ = 100 nM) and case 3 (K_m,d_ = 1 μM) compared to the base case. For all further analysis, K_m,d_ is assumed as 1 μM.

### The net charge on SM regulates the rate of IL-1α mediated cartilage degradation

**Fig 7:**
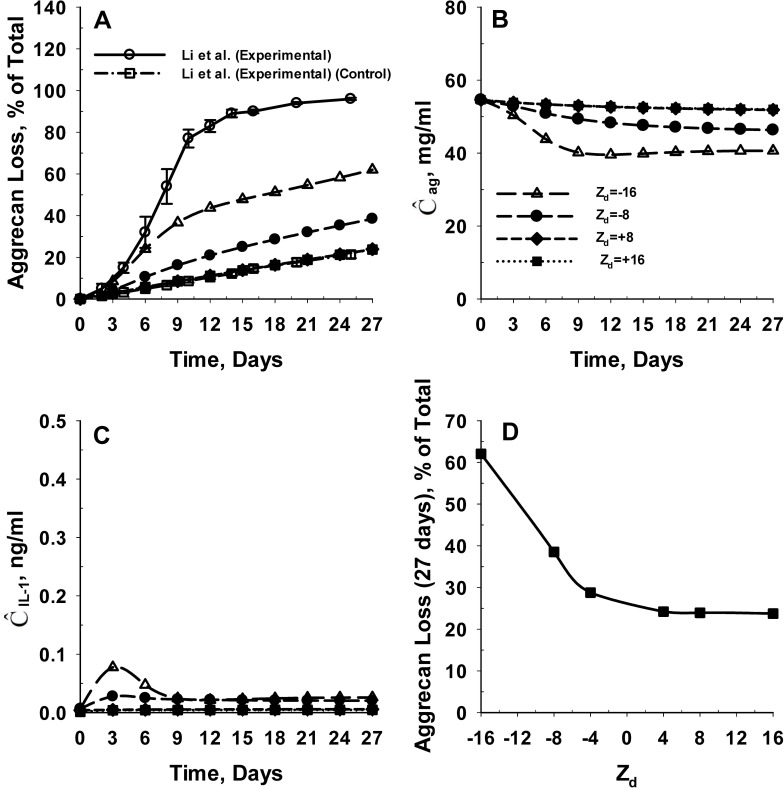
Effect of net charge of SM on inhibition of IL-1α mediated aggrecan catabolism in cartilage tissue. **Panel A** shows the effect of net SM charge (Z_d_) on the predicted rates of aggrecan losses from the cartilage tissue. The predicted rates of aggrecan loss are compared with *in vitro* aggrecan loss data from young bovine cartilage explants in the absence (control) and presence of IL-1α [14]. The model was simulated for net SM charge magnitudes (Z_d_) of -16, -8, +16 and +8. **Panel B** and **Panel C** show the temporal variation in the spatial average intact aggrecan and IL-1α concentration of the cartilage explant for the different cases simulated. **Panel D** shows the effect of net SM charge on the extent of aggrecan loss from the cartilage tissue at 27 days following initial exposure to IL-1α. The net charge on IL-1α is assumed to be -1 and the molecular weight of the SM is assumed to be 3 kDa. The dissociation constant related to binding of IL-1α to SM (K_m,d_) is assumed to be 1 μM.

Next we investigated the effect of the net SM charge on the inhibition of IL-1α mediated degradation of cartilage tissue. We investigated specific net charge magnitudes ranging from -16 to +16 (Z_d_ = -16, -8, +8 and +16, respectively). We assume a net charge of -1 on IL-1α (at physiological pH). The molecular weight of the SM is assumed to be 3 kDa. The simulation results appear in Fig 7. The results show that a positively charged SM can significantly reduce the rate of aggrecan loss from cartilage tissue compared to a negatively charged SM. An increase in the net SM charge magnitude from -16 to -8 causes a 38% reduction in aggrecan loss from the cartilage tissue at 27 days (Fig 7A and 7D). This is further reflected by the temporal variation in spatial average concentration of IL-1α (Ĉ_IL-1_) in the cartilage tissue, shown in Fig 7C. For net SM charge of -16, the spatial average IL-1α concentration of the explant increases from zero to a maximum value of 0.07 ng/ml at 3 days. Subsequently, the IL-1α concentration reduces to reach a steady state concentration of 0.02 ng/ml at 9 days. In contrast to net SM charge of -16, the spatial average IL-1α concentration of the explant reaches a steady state value of 0.02 ng/ml at 3 days corresponding to the net SM charge of -8.

The model also predicts that an increase in the net charge of SM from -8 to +4, eliminates aggrecan catabolism. The rate of aggrecan loss from the explant at net SM charge of +4 and above (Z_d_ = +8 and +16) (see Fig 7A and 7D), is consistent with reported *in vitro* aggrecan loss data from bovine cartilage explants cultured in the absence of IL-1α [14], that is, the spatial average aggrecan concentration in the cartilage tissue reduced by only 5% for positively charged SM (Fig 7B). As shown in Fig 7C, the spatial average concentration of IL-1α in the explant reduced by an order of magnitude for positively charged SM. Overall, these results show that positively charged SM may completely inhibit the IL-1α mediated aggrecan catabolism in cartilage tissue.

### Timing of SM administration on the inhibition of IL-1α mediated cartilage degradation

Our next step is to examine the effect of delaying SM administration on the inhibition of IL-1α mediated degradation of cartilage tissue. Hence we simulated five cases: (i) Case 1: the simultaneous administration of SM with IL-1α, (ii) Case 2: administration of SM 12 hours after initial exposure to IL-1α, (iii) Case 3: administration of SM 24 hours after initial exposure to IL-1α, (iv) Case 4: administration of SM 3 days after initial exposure to IL-1α and (v) Case 5: administration of SM 6 days after initial exposure to IL-1α. The time delay between the appearance of IL-1 and the administration of SM is denoted t_d_.

**Fig 8:**
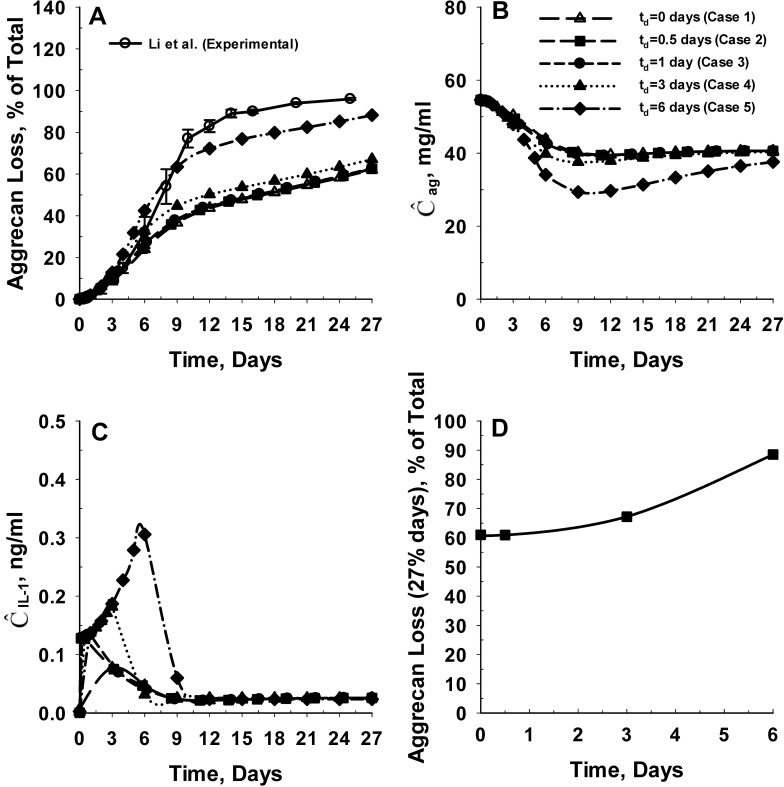
Effect of timing of SM administration on the inhibition of IL-1α mediated cartilage degradation. **Panel A** shows the effect of timing of SM administration post initial tissue exposure to IL-1α (t_d_) on the predicted rates of aggrecan losses from the cartilage tissue. The predicted rates of aggrecan loss are compared with *in vitro* aggrecan loss data from young bovine cartilage explants in the presence of IL-1α [14]. The model was simulated at five distinct values of t_d_ including 0 days (Case 1), 0.5 days (Case 2), 1 day (Case 3), 3 days (Case 4) and 6 days (Case 5). **Panel B** and **Panel C** show the temporal variation in the spatial average intact aggrecan and IL-1α concentration of the cartilage explant for the different cases simulated. **Panel D** shows the impact of SM administration timing (t_d_) on the extent of aggrecan loss from the cartilage tissue at 27 days for the different cases simulated. The net charge on IL-1α and SM are assumed to be -1 and -16, respectively. The molecular weight of the SM is assumed to be 3 kDa. The dissociation constant related to binding of IL-1α to SM (K_m,d_) is assumed to be 1 μM.

For these simulations we assume a net charge of -1 on IL-1α (at physiological pH) and -16 on the SM. Again the molecular weight of the SM is assumed to be 3 kDa. Simulation results appear in Fig 8. Surprisingly, the computational model results show that the SM can efficiently inhibit IL-1α mediated degradation of cartilage tissue as long as the SM is administered within 24 hours (1 day) following initial exposure of cartilage to IL-1α (Fig 8A). The average intact aggrecan and IL-1α concentration of the cartilage tissue are shown in Fig 8B and 8C. For case 1, the IL-1α concentration gradually increases with time to reach a maximum value of 0.07 ng/ml at 3 days. Following 3 days, the IL-1α concentration gradually reduces with time to reach a steady state value of 0.02 ng/ml at 9 days for cases 1, 2 and 3. The temporal variation in the spatial average concentration of intact aggrecan in the cartilage tissue is similar for cases 1, 2 and 3. However, intact aggrecan concentrations in the cartilage tissue were almost 10% lower if the SM is administered 3 days after initial exposure of the cartilage tissue to IL-1α (case 4).

For cases 2 and 3, the IL-1α concentration reaches its maximum value of 0.1 ng/ml over 3 to 12 hours and then drops to a value of 0.07 ng/ml at 3 days. However, the high IL-1α concentration in the cartilage tissue over 3 to 12 hours does not significantly impact the aggrecan degradation due to the time delay involved in the expression of aggrecanase following binding of IL-1α to IL-1 receptors [1, 86]. So if this is ‘shunt-down’ within 24 hours, aggrecanase production does not have the opportunity to become fully established. In contrast, administration of the SM after 72 hours (3 days) of exposure of the cartilage to IL-1α (Case 4) results in a 10% increase in aggrecan loss from cartilage tissue relative to cases 1, 2 and 3. The increase in aggrecan loss can be attributed to greater availability of IL-1α in the cartilage tissue between 3 to 6 days (Fig 8C), and so greater opportunity for production of aggrecanase to become established. We also see that that administration of the SM after 144 hours (6 days) of exposure of the cartilage to IL-1α (Case 5) results in a 45% increase in aggrecan loss from cartilage tissue in comparison to cases 1, 2 and 3 (Fig 8D), as aggrecanase production is comparatively high. Thus our results show that there exists a window of opportunity of 24 hours for administration of aggrecan catabolic inhibitors to effectively arrest the IL-1α mediated degradation of cartilage tissue before it becomes established.

### Competitive inhibition of IL-1α mediated cartilage degradation by SM

So far our model results are based on the assumption that the SM inhibits cartilage degradation by directly binding to IL-1α. However, there are several inhibitors of IL-1 signaling, which act by binding to IL-1 receptors (IL-1R) [2, 5, 74]. In other words, such inhibitors directly compete with IL-1 for binding to IL-1R [74]. In this section, we model competition between IL-1 and the SM for the IL-1 receptor.

In the absence of any inhibitor, the fraction of total IL-1R bound by IL-1α under equilibrium condition is given by [71]:

$$\frac{{\text{C}}^{*}}{{\text{C}}_{\text{IL}-1\text{R}}}=\frac{{\text{C}}_{\text{IL}-1}}{{\text{C}}_{\text{IL}-1}+{\text{K}}_{\text{m},\text{IL}1}}$$ (5)

However, the presence of an inhibitor capable of binding to IL-1R (e.g. the SM in our case) may significantly reduce the fraction of total IL-1R bound by IL-1α [87]. This can be mathematically represented as [71, 87]:

$$\frac{{\text{C}}^{*}}{{\text{C}}_{\text{IL}-1\text{R}}}=\frac{{\text{C}}_{\text{IL}-1}}{{\text{C}}_{\text{IL}-1}+{\text{K}}_{\text{m},\text{IL}1}\left(1+\frac{{\text{C}}_{\text{d}}}{{\text{K}}^{*}{}_{\text{m},\text{d}}}\right)}$$ (6)

where K*_m,d_ in Eq (6) represents the dissociation constant related to binding of the SM to IL-1R. Eq (6) is based on the assumption that the total concentration of IL-1α, SM and IL-1R in particular are constant [71] similar to our earlier model [1].

We implement the competitive inhibition of IL-1α mediated cartilage degradation in our model by substituting Eq (6) into the model governing equations of IL-1α and the stimulus responses representing the time delay in the expression of aggrecanase and MMP (S_1_ and S_2_). We assume a net charge of -1 for IL-1α and -16 for SM. The molecular weight of the SM is assumed to be 3 kDa. We selected the molecular weight and charge of the SM based on the reported molecular weight and isoelectric point of known osteoarthritis inhibitors including pentosan polysulfate (PPS) [12, 88, 89]. We simulated the model for four values of K*_m,d_ including 1 μM (Case 1), 1 nM (Case 2), 100 pM (Case 3) and 1 pM (Case 4). We selected these values based on the kinetic parameters related to the binding of IL-1α to IL-1R and the total IL-1R concentration (see Table 4). We compare the results from these cases with the predicted aggrecan loss profile in the absence of any chemical interaction between the SM and IL-1R (Base Case).

**Fig 9:**
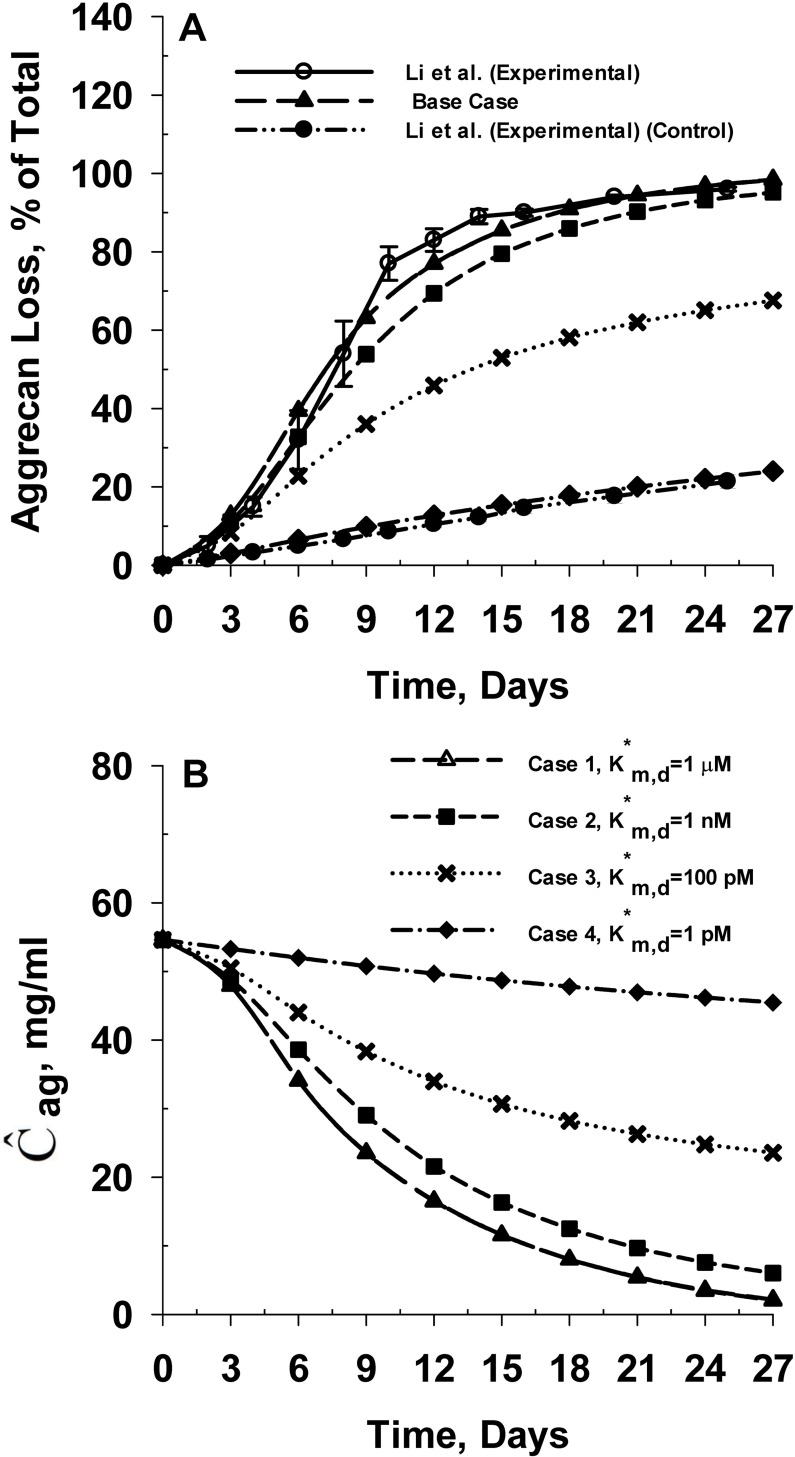
Effect of inhibition of IL-1α mediated degradation of cartilage tissue by binding of SM to IL-1 receptors (IL-1R). **Panel A** shows the effect of binding affinity of SM to IL-1R on the predicted rates of aggrecan losses from the cartilage tissue. The predicted rates of aggrecan loss are compared with *in vitro* aggrecan loss data from young bovine cartilage explants in the absence (control) and presence of IL-1α [14]. K*_m,d_ represents the dissociation constant related to binding of SM to IL-1R. The model was simulated at three distinct values of the dissociation constant (K*_m,d_) including 1 μM (Case 1), 1 nM (Case 2) and 1 pM (Case 3). The chemical interaction between the SM and IL-1R is not accounted for in the ‘Base Case’ scenario (no inhibition of IL-1α mediated cartilage degradation by the SM). **Panel B** shows the temporal variation in the spatial average intact aggrecan concentration of the cartilage explant for the different cases simulated. The net charge on IL-1α and SM are assumed to be -1 and -16 respectively. The molecular weight of the SM is assumed to be 3 kDa.

The simulation results are shown in Fig 9. The results show that the inhibition efficiency of the SM in mitigating IL-1α mediated aggrecan catabolism is negligible for cases 1 (K*_m,d_ = 1 μM) and 2 (K*_m,d_ = 1 nM) (Fig 9A). In fact competitive inhibition of cartilage degradation by the SM is completely ineffective at dissociation constant of 1 μM for binding of the SM to IL-1R, which stand in contrast to the small molecule binding directly to IL-1α. In order for the SM to be effective at large dissociation constants (e.g. 1 μM), it is necessary that the SM be positively charged. We find that (results not shown) the SM with a net charge of +16 could completely inhibit IL-1α mediated aggrecan catabolism at K*_m,d_ = 1 μM. Hence, the effectiveness of competitive inhibition of IL-1α mediated cartilage degradation is primarily dependent on Donnan partitioning of the SM from surrounding media to the cartilage tissue.

**Fig 10:**
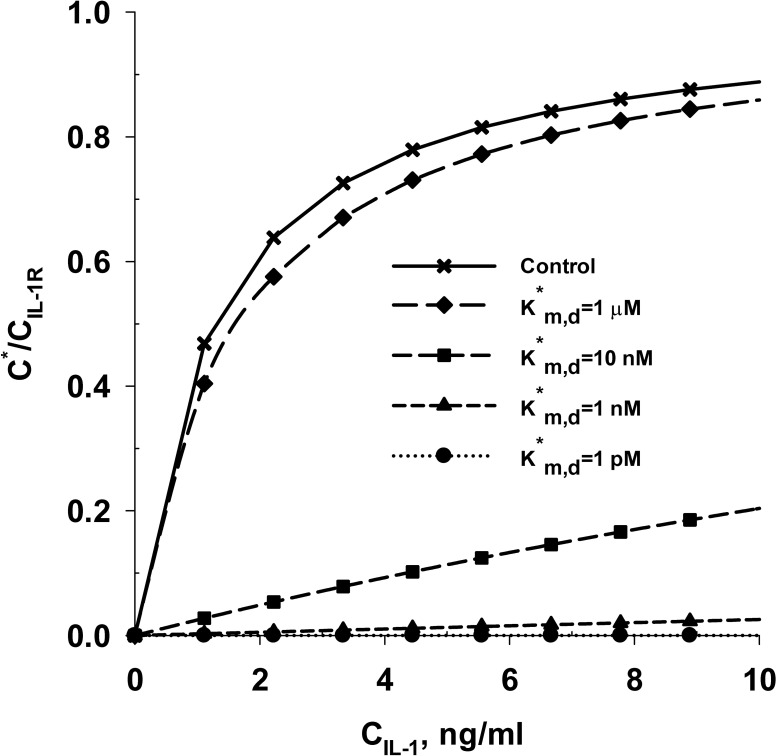
The fraction of total IL-1 receptors (IL-1R) bound by IL-1α under equilibrium condition in the presence of a SM (which is a competitive inhibitor of IL-1α). The parameter K*_m,d_ represents the dissociation constant related to binding of the SM to IL-1R. C_IL-1R_ represents the concentration of the total IL-1 receptors. C* represents the concentration of the IL-1-IL-1R complex formed due to binding of IL-1α with IL-1R. The concentration of the SM for the different binding constants (K*_m,d_) examined is set at a fixed value of 1 μg/ml. The ‘Control’ legend refers to the fraction of total IL-1 receptors (IL-1R) bound by IL-1α under equilibrium condition in the absence of SM.

For case 3 (K*_m,d_ = 100 pM), the aggrecan loss from the explant is reduced by about 33%. For case 4 (K*_m,d_ = 1 pM), IL-1α mediated aggrecan catabolism is completely inhibited, as the predicted aggrecan loss data from cartilage tissue is consistent with reported *in vitro* aggrecan loss data from bovine cartilage explants cultured in the absence of IL-1α [14, 26]. This is confirmed by the spatial average aggrecan concentration in the cartilage tissue (Fig 9B), which reduced by 96%, 90%, 57% and 17% at 27 days for cases 1 (K*_m,d_ = 1 μM), 2 (K*_m,d_ = 1 nM), 3 (K*_m,d_ = 100 pM) and 4 (K*_m,d_ = 1 pM), respectively. The results indicate that the ability of a molecule to competitively inhibit IL-1α mediated cartilage degradation depends strongly on its binding affinity with IL-1 receptors present on the chondrocyte surface, as otherwise concentrations of the small molecule need to be unrealistically high. Fig 10 shows that an increase in the binding affinity between SM and IL-1R (indicated by reduction in K*_m,d_) decreases the fraction of total IL-1R bound by IL-1α.

## Discussion

It is commonplace for various small molecules to be charged [12, 31, 90]. In the case of cartilage tissue, charge is particularly important because cartilage tissue has an unusually large negative fixed charge due to the high concentration of aggrecan [12, 41], which influences the uptake of charged molecules. The purpose of the computational models developed in this study is to assess the way an electrically charged small molecule influences cartilage degradation by binding to the inflammatory cytokine IL-1α. To do this, it is helpful to first consider Donnan partitioning of the SM between the synovial fluid and cartilage tissue alone. The results from our models (specifically the 1D model) have shown that in the absence of any size exclusion, Donnan partitioning may have a profound effect on the concentration distribution of any electrically charged species in the cartilage tissue and surrounding medium. Negatively charged molecules tend to be excluded from the cartilage tissue (known as ‘anion exclusion’), while positively charged molecules are drawn into the cartilage (known as ‘cation inclusion’). Assuming no interaction with the cartilage extracellular matrix, our models show that the time scales for the uptake and release of the SM are similar for any given specific net charge, as shown in Figs 4 and 5.

Specifically we observe that the time scales for the uptake and release of negatively charged molecules into a cartilage tissue of 1.5 mm thickness occurs on the order of hours. For example, the uptake and release times for a 3 kDa molecule with net charge of -16, -8 and -1 are 0.6, 0.8 and 1.1 hours, respectively. This is much the same for a 10 kDa small molecule. In contrast, the time scales for uptake and release of positively charged molecules are considerably longer, and range from hours to days. For example, the uptake (t_50,U_) and release (t_50,R_) times for a 3 kDa MW molecule with net charge of +1 is 3 hours, and for a 10 kDa MW molecule with a net charge of +8 is 2–3 days. However, the uptake and release times for a 10 kDa MW molecule with net charge of +16 is longer (t_50,U_ = 42 days and t_50,R_ = 72 days) in comparison to a 3 kDa MW molecule with the same net charge (t_50,U_ = 38 days and t_50,R_ = 62 days).

The MW dependent variation in the uptake and release times of molecules with net charge of +16 is due to the smaller diffusion coefficient of the 10 kDa molecule and the lower concentration of the drug in the synovial fluid (0.0001 moles/m^3^) [91]. Importantly, the total SM partitioning uptake for positively charged molecules by the cartilage tissue is about 1 to 3 orders of magnitude higher than that for negatively charged molecules of similar charge magnitude. Therefore, the partitioning storage of positively charged small molecules in cartilage ECM is substantial, which increases their duration of pharmacological action in cartilage tissue.

These SM uptake results are consistent with *in vitro* experiments designed to compare the uptake kinetics of NeutrAvidin with Avidin into young bovine cartilage tissue [12]. While these molecules are considerably larger than 3 kDa (MW = 66 kDa), and so size exclusion may apply to some degree [12], the total uptake of positively charged Avidin is reported to be approximately 400 fold higher in comparison to its neutral counterpart [12], which is broadly consistent with the theoretical predictions reported here. We also note in passing that Avidin was reported to demonstrate weak reversible binding with the cartilage ECM (K_m,ECM_ = 150 μM) [12]. The higher retention duration of positively charged molecules within the cartilage tissue may be advantageous therapeutically. For example, Avidin has been used as a carrier for drugs to modify cartilage degradation [12, 90].

The suitability of a drug to be used as an inhibitor of IL-1α mediated cartilage degradation depends on: (i) the concentration of the drug in the cartilage tissue, (ii) the dissociation constant (K_m,d_) related to binding of IL-1α to the drug and (iii) the uptake kinetics and the residence time of the drug in the cartilage tissue. We also note that for constant therapeutic effect, drug concentration may be ‘traded-off’ with the dissociation constant. It has been reported that IL-1α levels in synovial fluid increases significantly following traumatic injury to cartilage tissue [14, 92, 93]. The results presented here show that even small molecules with a very strong negative charge (MW = 3 kDa and Z_d_ = -16) can completely inhibit IL-1α mediated aggrecan catabolism provided it binds strongly to IL-1α with a dissociation constant of 10 nM or smaller (see Fig 6 and Table 5). Furthermore, even a SM with a net charge of -16 and a K_m,d_ of 1 μM can reduce cartilage degradation by about 30%, provided it is administered within a window of 24 hours following initial exposure of the cartilage tissue to IL-1α (see Fig 8 and Table 6). This highlights that a strong negative charge can be offset by a small dissociation constant. We note in passing that a dissociation constant of 10 nM is well above the reported dissociation constant of 30 pM for binding of IL-1α to the decoy IL-1α receptor, IL-1RII [18, 94, 95].

**Table 5:** Impact of IL-1α-SM binding kinetics for a SM (MW = 3 kDa, with a net charge of -16) on IL-1α mediated cartilage degradation rates.

K_m,d_ (nM)	Aggrecan Loss (27 days)
1	24.0%
10	25.0%
50	30.5%
100	35.3%
1000	62.0%
Experiment (IL-1α) [14]	96.0%
Experiment (Control) [14]	22.0%

**Table 6:** Impact of timing (t_d_) of SM (MW = 3 kDa, Z_d_ = -16 and K_m,d_ = 1 μM) administration on IL-1α mediated cartilage degradation rates.

t_d_ (days)	Aggrecan Loss (27 days)
0	62.0%
0.5	62.0%
1	62.0%
3	67.0%
6	88.2%

Clearly if the charge and concentration of a molecule in cartilage tissue is known or can be computed, then it is possible for our IL-1α induced cartilage degradation model to be used to infer the dissociation constant for binding of IL-1α to SM (K_m,d_). To illustrate the application of the model to experimental data, we consider Munteanu et al. [26], who reported on IL-1 induced degradation of cartilage (aggrecan loss), with and without pentosan polysulfate (PPS). PPS was used at 1 μg/ml in the bathing solution. If we make the reasonable assumption that the molecular weight of PPS was about 3 kDa and had a net charge of -16 (PPS is highly sulfated [96, 97]), and if we further assume that PPS binds directly to IL-1α, then the predicted aggrecan loss profiles are consistent with PPS binding to IL-1 with a K_m,d_ of 1 μM.

**Fig 11:**
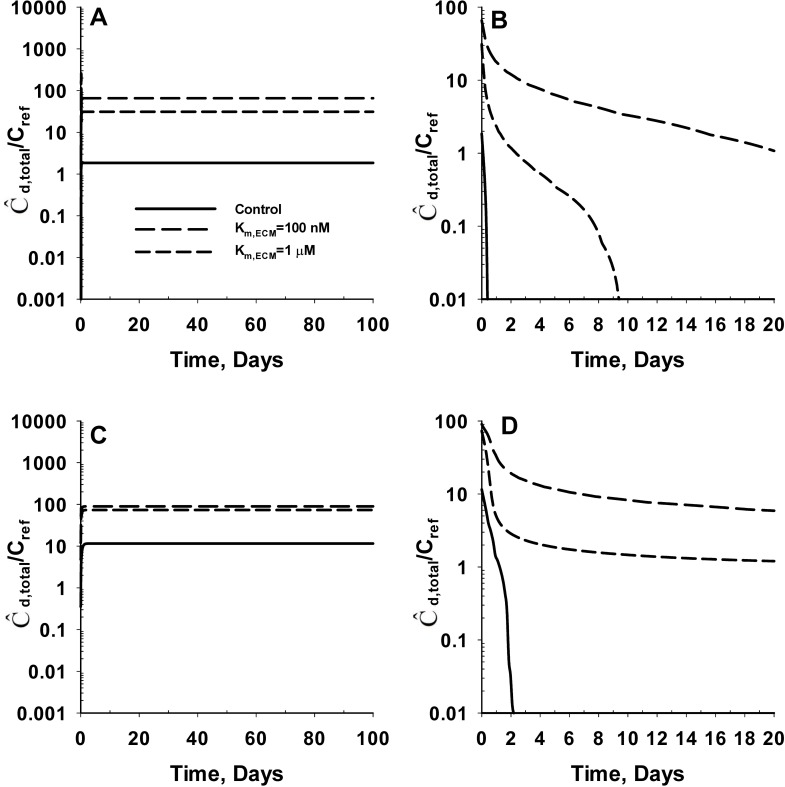
The *in vivo* uptake and retention of positively charged SM in cartilage tissue in the absence (‘Control’) and presence of SM binding to cartilage ECM. **Panel A** and **Panel B** shows the uptake and release kinetics of a small molecule with a net charge of +1 by the cartilage tissue. **Panel C** and **Panel D** shows the uptake and release kinetics of a small molecule with a net charge of +4 by the cartilage tissue. The parameter K_m,ECM_ represents the dissociation constant related to the binding of the small molecule (SM) to the cartilage ECM. Ĉ_d,total_ represents the spatial average total concentration of the drug in the cartilage tissue including the free drug concentration (Ĉ_d_) and the drug bound to the cartilage ECM (Ĉ_d,bound_). The bulk concentration of SM in the plasma (C_d,b_ or C_ref_) is assumed to be 1 μg/ml during the uptake process. The bulk concentration of SM in the plasma (C_d,b_) is assumed to be 0 μg/ml during the release process. The molecular weight of the SM is assumed to be 3 kDa for these simulations. The concentration of intact aggrecan throughout the cartilage tissue is set at a constant value of 60 mg/ml.

The binding coefficient required of small inhibitory molecules is considerably increased when the small molecules have a positive charge. Our results show that a small molecule that binds to IL-1α with a comparatively large dissociation constant of 1 μM, can completely inhibit IL-1α mediated aggrecan catabolism, provided the SM is positively charged (Fig 7 and Table 7). The complete inhibition of IL-1α mediated aggrecan catabolism under these conditions can be attributed to the higher concentration of SM in cartilage tissue due to cation inclusion. We note that a prolonged pharmacological action can be obtained when the small molecule has a large positive charge, as the time for release of the SM from cartilage tissue is extended (see Fig 5C). However, positively charged small molecules also take a longer time to achieve equilibrium with the cartilage tissue (see Fig 5A). Further, positively charged molecules may not only partition to cartilage ECM, but may also reversibly bind to the ECM [12]. Fig 11 shows our simulation results for the extended 1D model simulating the *in vivo* drug delivery scenario, taking into account the drug binding to the cartilage ECM. It is clear that binding of the SM to the cartilage ECM increases the steady-state average SM total concentration in the cartilage tissue by an order of magnitude. Additionally, the release times of the SM from the cartilage tissue are very significantly increased, which prolongs their duration of pharmacological action in cartilage tissue.

**Table 7:** Impact of SM (MW = 3 kDa, with a dissociation constant of 1 μM for binding to IL-1α) electrical charge (Z_d_) on IL-1α mediated cartilage degradation rates.

Z_d_	Aggrecan Loss (27 days)
-16	62.0%
-8	38.5%
-4	28.7%
+4	24.2%
+8	24.0%
+16	23.7%

For molecules that are cleared quickly from the bathing solution (e.g. small molecules in synovial fluid have a half-life of about 1–4 hours [40, 98–100]), a large positive charge will maximise uptake into the tissue in the time prior to clearance, as has been confirmed experimentally [12, 40]. Small molecules with net charge of +8, +10 and +16 partition ‘upwards’ from the synovial fluid to cartilage tissue with partitioning ratio (Ĉ_i,c_/Ĉ_i,f_) of approximately 6 within 4 hours as shown in Fig 5A, while the release may then take days. For example, after 4 hours of uptake, the time required for the average SM concentration to reach 50% of the initial average concentration during the release process (t_50,R_) are 2, 6 and 79 days corresponding to net SM charge of +8, +10 and +16 (results not shown). Hence, IL-1α inhibitor molecules are most effective in arresting cartilage degradation, when: (i) the molecule is small (preferable MW = 3–5 kDa) (ii) it has a large positive charge and (iii) it binds strongly to IL-1α and so has a small dissociation constant (e.g. 10 nM or below).

However, in addition to partitioning and binding of positively charged molecules to the cartilage ECM, other methods may be employed to sustain the inhibition of IL-1α mediated cartilage degradation including: (i) *conjugation* of OA drugs with positively charged carriers [12, 90] and (ii) *encapsulation* of OA drugs in particles and a particle based drug delivery system, which includes pastes [101, 102]. Conjugating OA drugs with positively charged carriers are reported to significantly increase their uptake and retention duration in cartilage tissue [12, 90]. Though these conjugated molecules are usually much larger than the SM reported here, these results are consistent with our model predictions (see Fig 5). The model developed here can be extended to model both conjugation and encapsulation.

The computational analysis to examine the effect of SM administration on the inhibition of cartilage degradation assumes that the SM binds with IL-1α to directly block the catabolic action of IL-1α. However, the SM may also interact with: (i) any of the molecules in the IL-1 and IL-1R families, and (ii) with downstream targets within the cell, or on molecules secreted by the cell [2, 74, 97]. For example, anakinra binds to the IL-1R [2]. This interaction may be incorporated into the present model by including competitive interaction between IL-1α and anakinra for the IL-1 receptor. Similarly it has been shown that PPS may exert its principal protective action in cartilage by inhibiting the activity of proteases (aggrecanases and MMPs) and not by binding to IL-1α directly [97, 103]. In this case, the model can be modified to incorporate the PPS dependent activity of aggrecanases and MMPs. In this way, the basic model presented here may be extended to include the large variety of possible SM interactions, depending on its intended purpose.

To illustrate how the model may be adjusted, we have examined the mechanism of competitive interaction between IL -1α and the SM for the IL-1 receptor. The results show that the binding coefficient required for a negatively charged SM (Z_d_ = -16) to competitively inhibit cartilage degradation ranges from 1–10 pM (K*_m,d_ = 1–10 pM). This is significantly lower compared to direct inhibition by binding of the SM to IL-1α (K_m,d_ = 1–10 nM) (see Figs 6 and 9). To view this in another way, for the same binding constant of 1 μM, the SM is much less effective at inhibiting cartilage degradation when it competitively binds to IL-1R than when it binds to IL-1 directly (compare Figs 6 and 9). The observed difference between the two inhibition mechanisms can be attributed to: (i) the strength of the binding coefficient related to the binding of IL-1α to its receptor (K_m,IL1_ = 72 pM) and (ii) the total IL-1 receptor concentration C_IL-1R_ = 0.7 nM, which is an order of magnitude higher than the maximum concentration of IL-1α (1 ng/ml). Hence the total fraction of IL-1 receptors bound by IL-1α drops from 86% to 3% as K*_m,d_ reduces from 1 μM to 1 nM as also illustrated in Fig 10.

However, there are some model limitations. The dissociation constant for binding between IL-1α and SM can be mathematically expressed as follows:

$${\text{K}}_{\text{m},\text{d}}=\frac{{\text{C}}_{\text{IL}-1}{\text{C}}_{\text{d}}}{{\text{C}}_{\text{comp}}}$$ (7)

In Eq (7) both IL-1α and SM are charged species. Assuming that the support medium/synovial fluid has a neutral pH, we can re-write Eq (7) within the cartilage tissue in terms of the synovial fluid/support medium concentrations of IL-1α and SM and the potential difference between synovial fluid/support medium and cartilage tissue (ΔV) as follows:

$${\text{K}}_{\text{m},\text{d}}=\frac{\left({\text{C}}_{\text{IL-}1,\text{f}}{\text{e}}^{\left(\frac{-{\text{Z}}_{\text{IL}-1}\text{F}\Delta \text{V}}{\text{RT}}\right)}\right)\left({\text{C}}_{\text{d},\text{f}}{\text{e}}^{\left(\frac{-{\text{Z}}_{\text{d}}\text{F}\Delta \text{V}}{\text{RT}}\right)}\right)}{{\text{C}}_{\text{comp}}}$$ (8)

The above equation indicates that Donnan partitioning may influence the distribution of both IL-1α and the SM. However, the valency (Z_i_) for the SM and IL-1α may also be influenced by the pH, which is itself also a function of Donnan partitioning. Hydrogen ions also partition due to the electrochemical gradient existing between the surrounding medium/synovial fluid and cartilage tissue. The pH of the surrounding medium/synovial fluid is around 7.2 [104]. It has also been reported that the pH of the cartilage tissue is approximately neutral at the surface, becoming somewhat acidic with depth (average pH is about 6.7 in normal cartilage). This is due to the increasing negative fixed charge with depth [38, 41, 105], together with the avascular nature of cartilage tissue, which favours anaerobic metabolism [78, 106]. Hence positively charged hydrogen ions are partitioned towards the negatively charged cartilage tissue. Using a Donnan partitioning ratio of 1.8 for species with net charge of +1 (Na^+^) as predicted by our model, we estimate the pH difference between the cartilage tissue and surrounding medium to be approximately 0.2 units. As this is reasonably small, the assumption of a constant pH of 7.0 for both the cartilage and surrounding medium appears to be a reasonable approximation for our analysis above. However, the assumption of Z_i_ being independent of pH could be relaxed for more accurate analyses.

## Conclusions

In this study, we have extended our previous model of IL-1α mediated degradation of bovine calf cartilage explants [1] to include the effects of (i) the negative fixed charge of the cartilage tissue and (ii) the Donnan partitioning of electrically charged species including IL-1α from the synovial fluid/support medium to the cartilage tissue. The goal of this study was to identify the key characteristics of an electrically charged small molecule (MW = 3–10 kDa), which can be used to effectively inhibit IL-1α mediated cartilage degradation. Our results show that even negatively charged small molecules (MW = 3 kDa, Z_d_ = -16) can inhibit IL-1α mediated cartilage degradation if administered within a stipulated timeframe following initial exposure of the cartilage tissue to IL-1α. Our analysis shows that even a small molecule with a negative charge of -16 binding directly to IL-1α with a dissociation constant of 1 μM, can preserve the cartilage collagen content and restore cartilage structural homeostasis if administered within a period of six days following initial tissue exposure to IL-1α. Furthermore, the depletion of aggrecan levels in cartilage tissue due to IL-1α exposure is completely restored in three weeks following administration of the small molecule (Fig 8B). However, when the small molecule competitively inhibits IL-1α by binding to its receptor, then to achieve a similar level of efficacy in protecting against cartilage degradation, the binding coefficient for the small molecule needs to be between 3 and 4 orders of magnitude smaller. This example highlights the relative efficacy of inhibiting signaling molecules rather than their receptors.

We also observed that positively charged small molecules can completely inhibit aggrecan catabolism (Fig 7). The suitability of positively charged small molecules for use as inhibitors of cartilage degradation can be attributed to (i) their higher uptake by cartilage tissue and (ii) their longer residence time in the cartilage tissue following uptake. However, even a strong negatively charged small molecule may be effective if the dissociation constant for binding to IL-1α is sufficiently small.

## Supporting Information

S1 Table Data related to Fig 2. (XLS)

S2 Table Data related to Fig 4. (XLS)

S3 Table Data related to Fig 5. (XLS)

S4 Table Data related to Fig 6. (XLS)

S5 Table Data related to Fig 7. (XLS)

S6 Table Data related to Fig 8. (XLS)

S7 Table Data related to Fig 9. (XLS)

S8 Table Data related to Fig 10. (XLS)

S9 Table Data related to Fig 11. (XLS)
